# Single‐cell landscape of the tumour immune microenvironment in human gynaecologic malignancies

**DOI:** 10.1002/ctm2.70538

**Published:** 2025-11-23

**Authors:** Simin Yin, Sen Li, Mengyan Tu, Junfen Xu

**Affiliations:** ^1^ Zhejiang University School of Medicine Hangzhou Zhejiang China; ^2^ Zhejiang Key Laboratory of Precision Diagnosis and Therapy for Major Gynecological Diseases Women's Hospital Zhejiang University School of Medicine Hangzhou Zhejiang China; ^3^ Department of Gynecologic Oncology Women's Hospital Zhejiang University School of Medicine Hangzhou Zhejiang China

**Keywords:** cervical cancer, endometrial cancer, single‐cell RNA sequencing, tubo‐ovarian cancer, tumour immune microenvironment

## Abstract

**Background:**

The immune microenvironment of the three most common gynaecological malignancies—tubo‐ovarian cancer, endometrial cancer and cervical cancer—has not been systematically studied, limiting clinical application.

**Methods:**

This study analyses 272 389 *CD45*+ immune cells by integrating publicly available single‐cell RNA sequencing (scRNA‐seq) data from 111 tumour and non‐malignant tissue samples. We identified distinct subsets within immune cells: 11 for monocytes/macrophages, six for CD4 T cells, eight for CD8 T cells and five for B cells, detailing their distribution, prevalence and distinct functions.

**Results:**

A pro‐angiogenic macrophage subset linked to NF‐κB signalling was associated with worse clinical outcomes and an interferon‐primed macrophage subset correlated with improved survival by recruiting T cells through CXCL9/10/11 secretion, as confirmed by multi‐colour immunohistochemistry. T cells exhibited dynamic roles in tubo‐ovarian cancer, with CD8 Tex cells contributing to immune dysfunction and poor prognosis, while CD8 Trm cells in early‐stage tumours supported immune surveillance. Additionally, we identified co‐stimulatory and co‐inhibitory receptor interactions and classified distinct B cell subsets with varying prognostic implications.

**Conclusions:**

This comprehensive analysis of the tumour immune microenvironment in gynaecological malignancies provides new insights into immune cell composition and function offering potential for optimising immunotherapies and improving clinical outcomes in these cancers.

## Graphical abstract

This study presents a comprehensive single‐cell atlas of the immune landscape in gynaecological tumours. Angio‐Mac, driven by NFKB1, was associated with worse clinical outcomes. IFN‐Mac_CXCL9 improved survival by recruiting T cells through CXCL9/10/11 secretion. T cells experienced a shift from anti‐tumour to pro‐tumour during the progression of cancer.

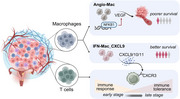

**Abbreviations**
DEG: differentially expressed gene
GO: Gene Ontology
HGSOC: high‐grade serous ovarian cancer
SCENIC: Single‐Cell regulatory Network Inference and Clustering
scRNA‐seq: single‐cell RNA sequencing
TAM: tumour‐associated macrophage
TF: transcription factor
TIME: tumour immune microenvironment
TME: tumour microenvironment

## 1 INTRODUCTION

Cervical, ovarian and endometrial cancers represent the three most prevalent gynaecological malignancies. Cervical cancer is the fourth most diagnosed cancer and the third leading cause of cancer‐related deaths in women. Annually, cervical cancer is responsible for over 660 000 newly cases worldwide.^1^ Human papillomavirus (HPV) infection, particularly persistent infection, is a well‐established cause of cervical cancer, especially squamous cell carcinoma,^2^ which underpins the rationale for prophylactic HPV vaccination. Ovarian cancer, the most lethal gynaecological malignancy, causes approximately 210 000 deaths each year,^1^ with high‐grade serous ovarian cancer (HGSOC) accounting for 80% of these fatalities.^3^ Ovarian cancer is typically diagnosed at advanced stages, often with intraperitoneal spread at the time of diagnosis, and it has a poor prognosis due to its propensity for recurrence. Emerging evidence suggesting that ovarian cancer may originate from the fallopian tube^4^, ^5^, ^6^ has led us to expand our research to include tubo‐ovarian cancer. Endometrial cancer, often presenting as abnormal uterine bleeding in postmenopausal women, significantly impacts quality of life. This cancer is increasingly associated with metabolic disorders, such as obesity, diabetes and hypertension, and its incidence continues to rise.^7^, ^8^, ^9^, ^10^

Recent advancements in single‐cell RNA sequencing (scRNA‐seq) have led to numerous studies characterising the tumour microenvironment (TME) in gynaecological malignancies. Hornburg et al.^11^ introduced tumour immune phenotypes (TIPs) in ovarian cancer, categorising them into three types: immune‐infiltrated, immune‐excluded and immune‐desert. Similarly, a two‐meta‐program model involving malignant epithelial cells interacting with different TIPs was identified in cervical squamous cell carcinoma, elucidating the limited efficacy of immune checkpoint blockade therapy in this cancer type.^12^ In our previous studies, we identified molecular markers in tumour cells and explored metabolic alterations in cancer‐associated fibroblasts and macrophages in HGSOC, as well as novel therapeutic targets such as TIGIT+ T cells^13^ and S100A9+ tumour cells.^14^ Cassier et al.^15^ reported that the blockade of Netrin‐1 could enhance CD8 T cell‐tumour cell interactions and reduce pro‐tumour M2‐like macrophages in endometrial cancer, providing evidence for targeted therapies. These insights have greatly deepened our insight into the mechanisms driving the progression, metastasis and drug resistance of tumours. Despite these advances, several critical aspects of gynaecological cancer research remain unexplored, particularly in terms of the tumour immune microenvironment (TIME). Current studies still lack a comprehensive and systematic characterisation of the immune ecosystem. Furthermore, the limited success of immunotherapy in ovarian cancer,^16^, ^17^, ^18^ the lack of definitive immunotherapy guidelines for endometrial cancer, and the variable response to PD‐1/PD‐L1 therapies in cervical cancer underscore the urgent need for improved therapeutic targets and more precise treatment response predictions. Thus, it is a pressing need to further investigate the TIME in gynaecological malignancies.

This study combined publicly accessible scRNA‐seq datasets to create a comprehensive immune profile of the TME in tubo‐ovarian, endometrial and cervical cancers. We identified six CD4 T cell subtypes, eight CD8 T cell subtypes, five B cell subtypes and 11 monocyte/macrophage subtypes. Analysis of immune cell compositions across various cancer stages and non‐malignant tissues revealed two macrophage subsets: a pro‐angiogenic subset linked to poor survival via the NF‐κB signalling pathway and an interferon‐primed subset associated with improved survival, capable of recruiting T cells through CXCL9/10/11–CXCR3 interactions. The transformation of T cells from anti‐tumour to pro‐tumour was also discovered. Our study provides a comprehensive and systematic characterisation of the TIME in gynaecological malignancies, offering new insights into immune cell characterisation, highlighting potential therapeutic targets for improving immunotherapy outcomes.

## 2 MATERIALS AND METHODS

### 2.1 Collection and processing of scRNA‐Seq datasets

scRNA‐Seq datasets were collected from publicly available resources (Table S1). Data preprocessing steps included quality control and normalisation and the integration of datasets from different sources. The doublets were identified and filtered using the scDblFinder package (v.1.12.0). Cells expressing 200 to 10 000 genes with mitochondrial UMI fractions under 20% were retained, excluding mitochondrial genes from further analysis. The Seurat package (v4.4.0) was employed for data normalisation and scaling. Principal component analysis was carried out on the 2000 most variable genes, followed by batch‐effect correction using Harmony (v1.1.0). The resulting embeddings were visualised with UMAP, and graph‐based clustering was applied to define cell populations. Differentially expressed genes between clusters were identified using the Wilcoxon rank‐sum test in Seurat, applying criteria of log2FC > .25, adjusted *p*‐value < .05 and min.pct > .1. Gene Ontology enrichment was assessed through clusterProfiler (v.4.11.1),^19^ considering pathways with adjusted *p* < .05. A Bonferroni correction was used for multiple comparisons. Additional methods for the single‐cell analytical procedures are provided in the Supporting Information.

### 2.2 Survival analysis

Survival analysis utilised clinical and bulk RNA sequencing data from The Cancer Genome Atlas (TCGA). Signature genes derived from distinct cell clusters or specific genes were utilised for prognostic evaluation.^20^ The analysis utilised datasets from TCGA‐OV (ovarian cancer), TCGA‐CESC (cervical cancer) and TCGA‐UCEC (endometrial cancer), accessible via the GDC Data Portal (https://portal.gdc.cancer.gov/).

For each set of marker genes, the following steps were carried out: (1) *Marker gene selection*: Genes with log2FC > .25 were selected. (2) *Calculation of signature gene scores*: The relative expression of signature genes for each cluster was assessed using the calculate_sig_score function from IOBR (v.0.99.8).^21^ This involved converting gene expression counts to transcripts per million values and applying the *z*‐score method. (3) *Patient stratification*: Patients were dichotomised into high‐ and low‐expression cohorts using the log‐rank test‐determined optimal threshold. Survival differences were illustrated using Kaplan–Meier curves created with survminer (v0.4.9). Multivariable Cox regression analyses were further performed to assess the independent prognostic value of high/low expression groups, with adjustment for patient age, disease stage and tumour grade.

### 2.3 Cell line culture and CRISPR–Cas9‐mediated gene knockout

THP‐1 and Jurkat T cells were cultured in RPMI 1640 medium with 10% FBS and 1% penicillin/streptomycin; THP‐1 cells also received 0.5 mM β‐mercaptoethanol.

Stable *NFKB1* and *CXCL9* knockout cell lines were generated using a lentiviral CRISPR–Cas9 system. The single‐guide RNA (sgRNA) sequences targeted by NFKB1, CXCL9#1, #3 CRISPR are CCTGTTGGCAGTGCCATCTG, CAGCGACCCTTTCTCACTAC and GCGACCCTTTCTCACTACTG, respectively.

The sgRNA oligos for target genes were annealed and cloned into lentiCRISPR v2 vector (52961; Addgene) digested by BsmBI‐v2 (R0739; NEW ENGLAND Biolabs). HEK293 cells were co‐transfected with pMD2.G (12259; Addgene), psPAX2 (12260; Addgene) and LentiCRISPR v2 vector, using Lipofectamine 3000 (L3000015; Thermo Fisher Scientific) for 48 h to produce lentivirus. THP‐1 cells underwent lentiviral infection and subsequent selection with 2 µg/mL puromycin (HY‐B1743; MCE) for 7 days.

### 2.4 Chemotaxis assays

THP‐1 cells underwent M0 macrophage differentiation via phorbol 12‐myristate 13‐acetate (PMA; 100 ng/mL; Sigma–Aldrich) over a 24‐h period, followed by polarisation to M1 macrophages with 100 ng/mL LPS (Sigma–Aldrich) and 20 ng/mL IFN‐γ (PeproTech) for 48 h. For the chemotaxis assay, 1 × 10^6^ Jurkat T cells, labelled with 10 µM Cell Proliferation Dye eFluor™ 450 (Invitrogen), were suspended in 200 µL RPMI 1640 medium with 2% foetal bovine serum and placed in the top chamber of 8.0 µm pore Transwell inserts (Corning).THP‐1‐derived M1 macrophages (1 × 10^6^) were seeded at the bottom chamber. Jurkat T cell migration from the top to the bottom chamber was quantified after a 4‐h incubation at 37°C with 5% CO_2_ using flow cytometry and Precision Count Beads™ (424902; Biolegend). IL‐2 levels in co‐culture supernatants collected after 24 h were measured by ELISA (ABclonal RK00002).

### 2.5 IKK treatment and measurement of VEGFA

M2 macrophages were derived from M0 cells via IL‐4 (20 ng/mL, 200‐04; Sigma–Aldrich) and IL‐13 (20 ng/mL, 200‐13; PeproTech) treatment for 48 h, followed by treatment with the IKK inhibitor BAY 11‐7082 (20 µM, HY‐13453, MCE) for another 48 h. VEGFA levels in the culture supernatants were measured via ELISA (ABclonal RK00023), and total RNA was extracted for qPCR analysis.

### 2.6 Tissue sample collection

Formalin‐fixed, paraffin‐embedded (FFPE) primary tissue samples were obtained from pathologically confirmed HGSOC patients undergoing primary surgery at the Women's Hospital, Zhejiang University. Detailed information on the specimens is provided in Table S3.

### 2.7 Multi‐colour immunohistochemistry staining

The FFPE tissue samples were sectioned into 4‐µm‐thick slides, followed by deparaffinisation. Multi‐colour immunohistochemistry (mIHC) staining was performed with a multi‐colour Immunofluorescence Kit (Akoya Biosciences) following the manufacturer's instructions. Slides were boiled in antigen retrieval solution for 15 min, then blocked and incubated with the primary antibodies: anti‐CD68 (1:400, 76437; Cell Signaling Technology), anti‐NFKB1 (1:100, A11160; ABclonal) and anti‐VCAN (1:100, A19655; ABclonal) for Angio‐Mac and anti‐CXCL9 (1:100, Ab9110; ABcam) and anti‐PITX1 (1:100, 10873‐1‐AP; Proteintech) for IFN‐Mac_CXCL9 at 37°C for 1 h. This was followed by incubation with HRP‐conjugated secondary antibodies. Signals were visualised with Opal TSA reagents and counterstained with DAPI. The slides were then analysed using a confocal laser‐scanning microscope (STELLARIS5; Leica).

### 2.8 Statistical analysis

scRNA‐seq analysis was performed using R version 4.2.2. Gene expression among distinct cell types or groups was assessed via the Wilcoxon rank‐sum test. Survival analysis *p* values were derived using the log‐rank test. Cell proportions were compared using Graphpad 10.1.2, employing non‐parametric tests such as the Mann–Whitney test for pairwise comparisons and the Kruskal–Wallis test for multi‐group comparisons. Statistical analysis of ELISA and qPCR data employed either Student's *t*‐test or one‐way ANOVA for group comparisons. A *p* value below.05 was deemed statistically significant.

## 3 RESULTS

### 3.1 Construction of a single‐cell transcriptome atlas of immune cells in gynaecologic cancers

**FIGURE 1:**
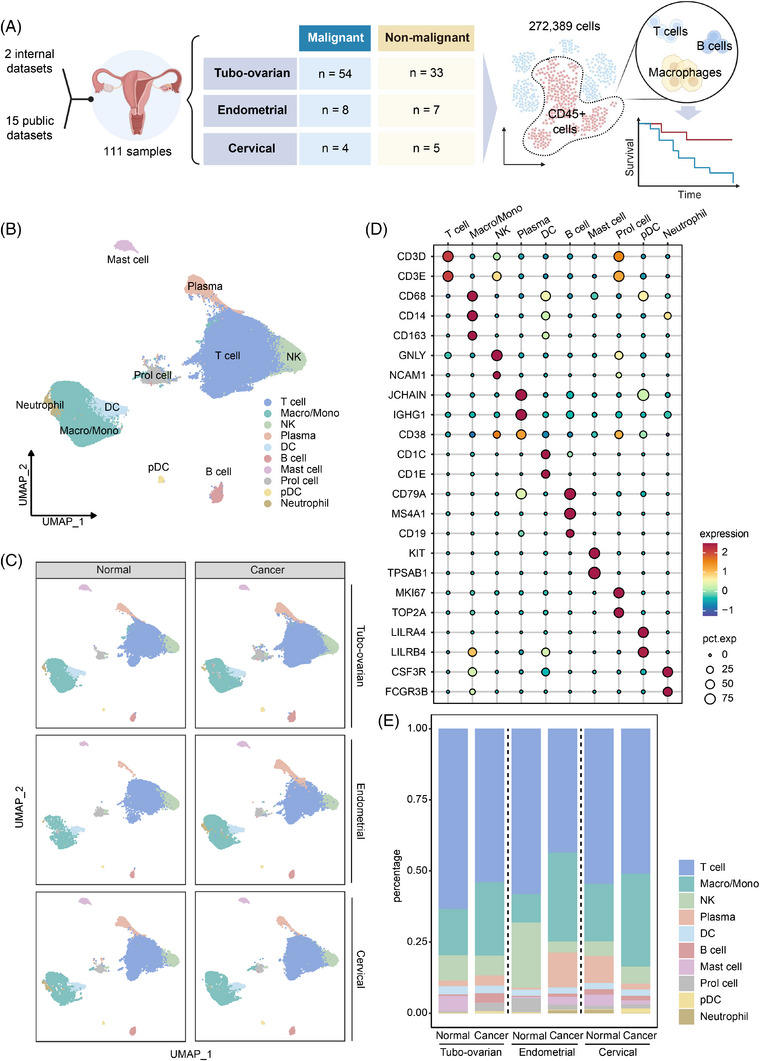
Identification of various immune cell types in gynaecologic malignancies. (A) Workflow showing the collection and processing of specimens of tumours and non‐malignant tissues for scRNA‐seq analysis. (B) UMAP plot depicting main immune cell types in tumours and control tissues with distinct colours assigned to each cell type. (C) UMAP plot depicting immune cell types in tumours of tubo‐ovarian cancer, endometrial cancer, cervical cancer and their non‐malignant counterparts. (D) Dot plots demonstrating the expression levels of specific marker genes in different cell types. The dot size indicates the proportion of cells expressing the respective marker gene, while the colour spectrum represents the average expression levels of the marker genes. (E) Bar plot showing proportion of each cell type in tumours of tubo‐ovarian cancer, endometrial cancer, cervical cancer and their non‐malignant counterparts.

We compiled scRNA‐seq data from 111 samples across existing datasets to create a single‐cell transcriptome atlas of the TIME in the three primary gynaecologic cancers (Figure 1A). The samples comprised 54 from tubo‐ovarian cancer patients, eight from endometrial cancer patients and four from cervical cancer patients. Additionally, non‐malignant samples from the ovary/fallopian tube (33 samples), endometrium (seven samples) and cervix (five samples) were also included for comparison. The tumour samples were diverse in terms of their anatomical sites, pathology and clinical stages, ensuring the broader applicability of our analysis to gynaecologic malignancies. For the purpose of this study, tumour samples were categorised into early or late stages based on the FIGO classification, or by their metastasis and recurrence status in the absence of staging information. Among these, 18 samples are internal, having been sequenced for previous studies.^13^, ^22^ A summary of the datasets used is provided in Table S1.

After integration and stringent quality control, the datasets yielded a final set of 272 389 *CD45*+ immune cells for analysis (Figure S1A–C). Unsupervised clustering was performed on these immune cells, with batch effects corrected using Harmony. The clustering results were visualised using UMAP. Using canonical cell markers, we identified 10 major immune cell linages (Figure 1B, D): T cells (marked by *CD3D* and *CD3E*), natural killer (NK) cells (*GNLY*, *NCAM1*), plasma cells (*JCHAIN*, *IGHG1*, *CD38*), B cells (*CD79*, *MS4A1*, *CD19*), plasmacytoid dendritic cells (pDCs; marked by *LILRA4* and *LILRB4*), mast cells (*KIT*, *TPSAB1*), macrophages and monocytes (macro/mono; marked by *CD68*, *CD14* and *CD163*), dendritic cells (DC; marked by *CD1C* and *CD1E*), a small cluster of neutrophils (*CSF3R*, *FCGR3B*) and proliferating cells (Prol cells; marked by *MKI67* and *TOP2A*). The immune cell subsets were well distributed across the integrated data (Figure S1D), and the composition of immune cell types showed similar patterns across all three cancer types and their non‐malignant counterparts (Figure 1C).

We further examined the constitution of immune cell types in the dataset. Figure 1E illustrates that T cells predominated among immune cell types in both cancerous and normal groups, comprising almost half of the tumour‐infiltrating immune cells. Macrophages and monocytes were the second most prevalent immune cells, except in the normal endometrium, where uterine NK cells significantly regulate the endometrial immune environment throughout the menstrual cycle. B cells were generally less abundant than plasma cells across all groups, except in the tubo‐ovarian and cervical cancer groups, aligning with Laumont et al.'s findings.^23^ DCs were uniformly distributed across the six groups, while pDCs were predominantly observed in the tubo‐ovarian and cervical cancer samples.

### 3.2 Heterogeneity of macrophage phenotypes in the TIME

**FIGURE 2:**
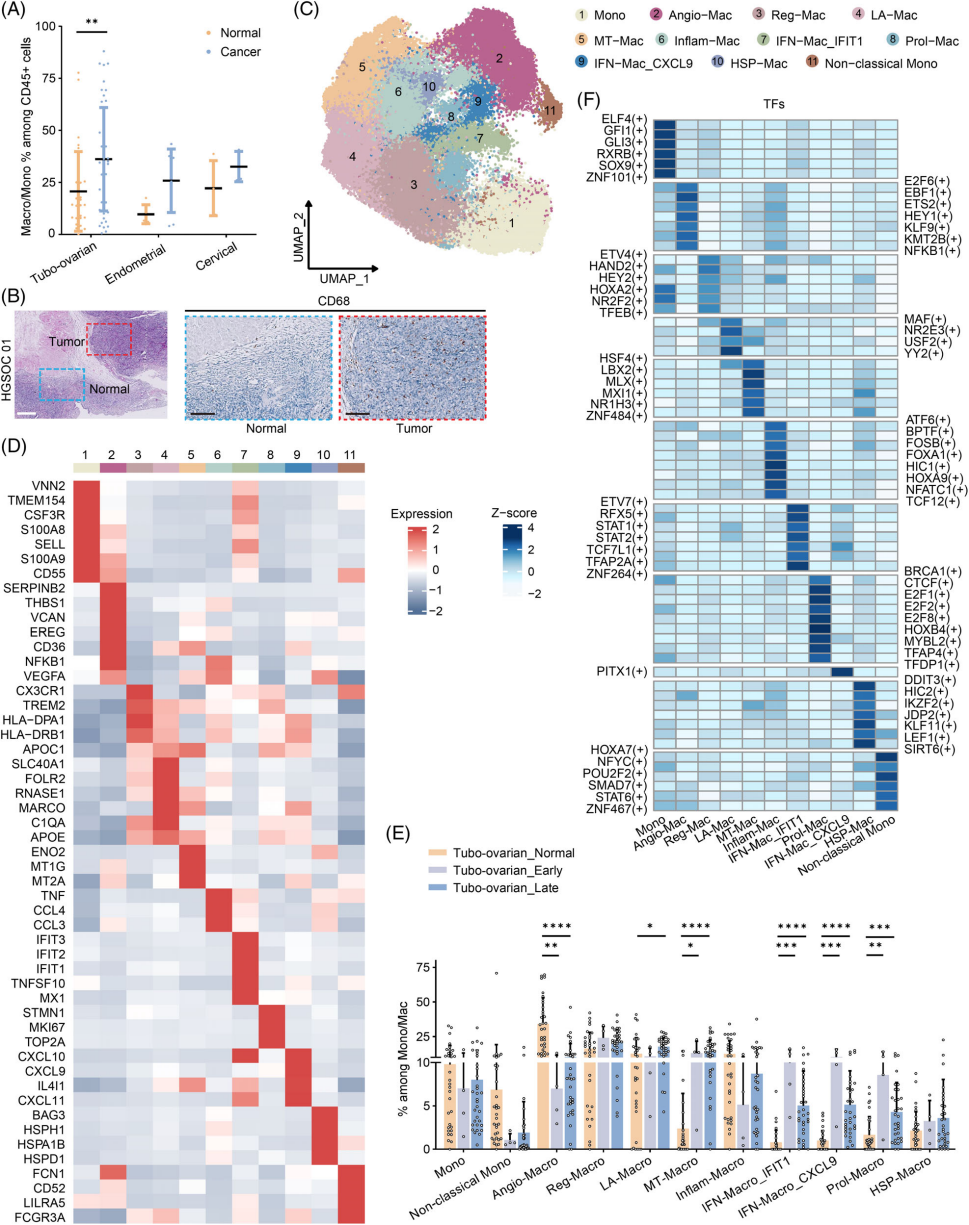
Cell clustering and annotation of monocytes and macrophages in gynaecologic malignancies. (A) Percentages of macro/mono in tumours and control tissues. The p values were calculated by Mann–Whitney test. (B) Representative images of H&E and IHC staining for CD68 in tumour and normal adjacent tissue in HGSOC. Scale bars, 250 µm (H&E) and 100 µm (IHC). (C) UMAP plot displaying the re‐clustering macro/mono with distinct colours assigned to each subset. (D) Heatmap depicting the re‐clustering of macro/mono with distinct signature genes. (E) Bar plots showing the fraction of each cluster relative to the total macro/mono count in control tissues and early‐stage or late‐stage tubo‐ovarian cancer. The p values were calculated by Kruskal–Wallis test. (F) Heatmap showing specific TFs for each subset of macro/mono. The activity of TFs was represented by scaled AUCell score. ****p < 1.0e−4, ***p < 1.0e−3, **p < .01, *p < .05.

Tumour‐associated macrophages (TAMs) are a major element of the TIME. We observed an increased proportion of macro/mono in tumour samples compared with non‐malignant samples, with this difference being statistically significant only in the tubo‐ovarian cancer group (Figure 2A). We performed CD68 immunohistochemistry on FFPE samples from HGSOC patients, which further validated the preferential distribution of macrophages in tumours verses normal adjacent tissue (Figures 2B and S2).

Macrophages are well documented for their pro‐tumour functions, contributing to tumour progression and invasion.^24^, ^25^, ^26^ To investigate the subsets of macro/monos, we identified that some genes were not sequenced in certain samples, which disrupted the subclustering process (Figure S3A–E). To address this, we filtered out the genes that were not consistently sequenced across all samples, retaining only those present in all samples. This allowed us to re‐cluster the cells into 11 distinct subsets (Figures 2C,D and S3F,G).

While previous studies have often classified macrophage subsets based on marker genes, we adopted a model established by Ma et al.^27^ for more detailed categorisation. Cluster 1 and 11 were classified as monocytes and non‐classic monocytes (or *CD16*+ monocytes), respectively. Cluster 2, marked by the expression of angiogenic factors like *VCAN*, *THBS1* and increased *VEGFA*, was identified as pro‐angiogenic macrophages (Angio‐Mac). Cluster 3 showed elevated levels of *CX3CR1*, *TREM2* and genes associated with antigen presentation (e.g., *HLA‐DPA1*/*DRB1*) and was designated as immune regulatory macrophages (Reg‐Mac). Cluster 4, marked by lipid‐related genes (e.g., *APOE*, *APOC1*), was classified as lipid‐associated macrophages (LA‐Mac), which also expressed signatures of resident‐tissue macrophages (e.g., *MARCO*, *FOLR2* and *SLC40A1*). Cluster 6, enriched for inflammatory cytokines (*TNF*, *CCL3* and *CCL4*), was termed inflammatory cytokine‐enriched macrophages (Inflam‐Mac). We identified two distinct interferon‐primed macrophage subsets (IFN‐Mac). Cluster 7 showed expression of *IFIT1/2/3*, while Cluster 9 expressed *CXCL9/10/11*. The remaining three clusters were designated as MT‐Mac, Prol‐Mac and HSP‐Mac based on their gene signatures related to metallothionein, cell proliferation and heat shock proteins, respectively.

Further analysis revealed the distribution of these 11 macrophage subsets in tumours and non‐malignant tissues. Angio‐Mac was predominantly found in non‐malignant ovaries and fallopian tubes (Figure 2E), indicating its distinct function in normal tissue compared with tumours. Both IFN‐Mac subsets were significantly enriched in tubo‐ovarian cancer, regardless of tumour stage (Figure 2E). However, no significant difference was observed in endometrial and cervical cancer (Figure S4A,B). Additionally, MT‐Mac was found to be increased in both tubo‐ovarian and endometrial cancer, likely as a result of oxidative stress in the TME. To further validate the reliability of our clustering, we performed subsampling‐based robustness checks and most subsets exhibited high Jaccard consistency (Figure S4C). Additionally, we repeated the clustering process to two independent cohorts^28^, ^29^ (Table S2) for endometrial and cervical cancers, and the majority of clusters, including monocytes, Angio‐Mac, LA‐Mac, Reg‐Mac, Infam‐Mac, HSP‐Mac and IFN‐Mac, were successfully identified (Figure S5), further supporting the reproducibility of the annotation system in gynaecologic malignancies.

To pinpoint the transcription factors (TFs) crucial for macro/mono subset formation, we utilised Single‐Cell Regulatory Network Inference and Clustering (SCENIC) analysis. Distinct TFs were identified for each cluster (Figure 2F). Notably, members of the STAT family, including STAT1 and STAT2, emerged as the top TFs associated with IFN‐Mac_IFIT1 activation, consistent with their established role in mediating responses to interferon signalling.^30^, ^31^ Utilising the ChIP‐Atlas database (https://chip‐atlas.org/), multiple ChIP‐seq datasets derived from monocytes confirmed that STAT1 binds to key marker genes of IFN‐Mac_IFIT1 (Figure S6A). Motif enrichment analysis on IFN‐Mac_IFIT1 signatures through RcisTarget also confirmed enrichment for two STAT1‐binding motifs: metacluster_2.6 (NES = 8.24, AUC = .103) and metacluster_2.7 (NES = 4.99, AUC = .0737) (Figure S6B). Additionally, PITX1 was identified as the key TF for IFN‐Mac_CXCL9, while NFKB1 was implicated as the central TF for Angio‐Mac.

### 3.3 IFN‐Mac_CXCL9 and Angio‐Mac are correlated with differing prognosis in cancer

**FIGURE 3:**
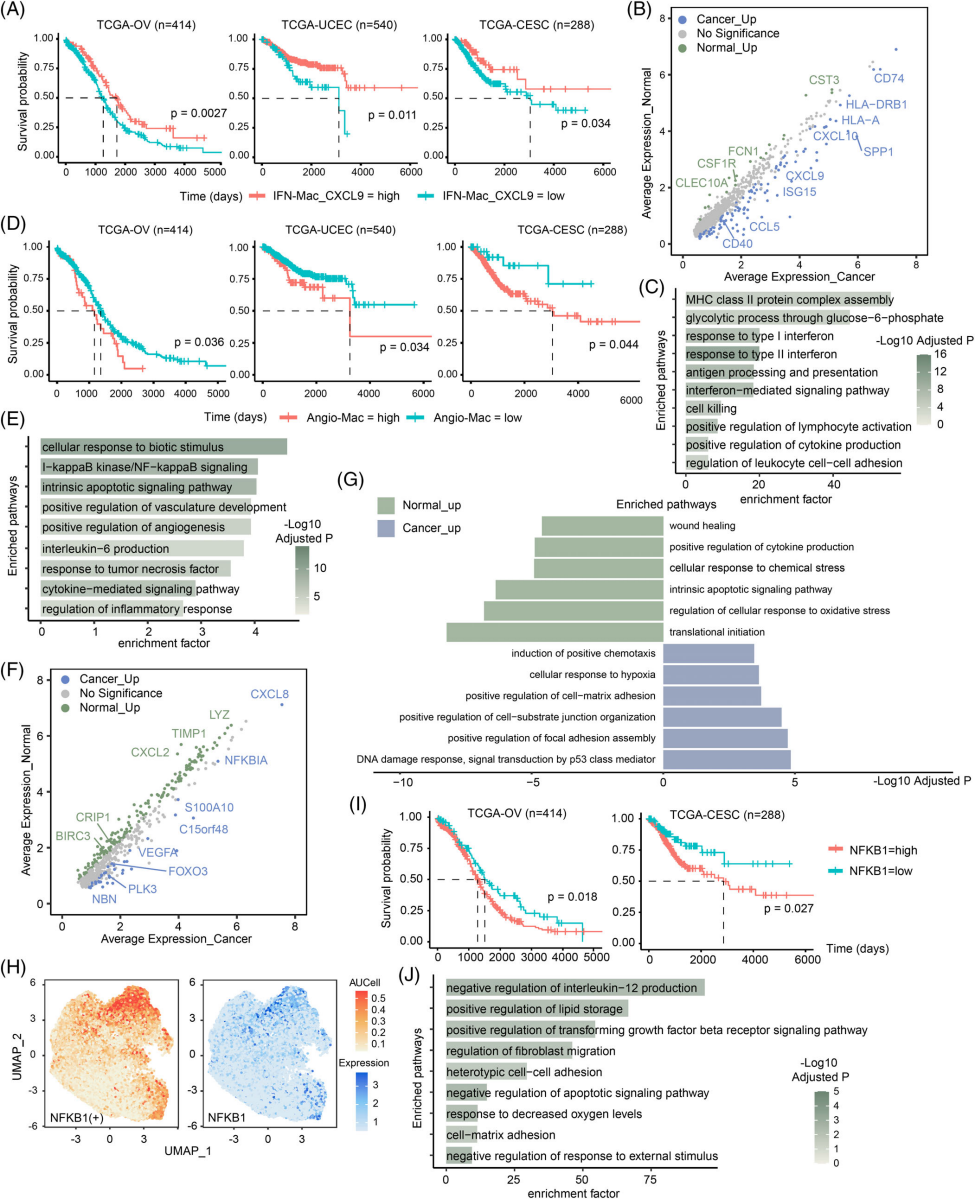
Characteristics of IFN‐Mac_CXCL9 and Angio‐Mac. (A) Kaplan–Meier curves for overall survival generated from TCGA cohorts of ovarian, endometrial and cervical cancer, demonstrating significant prognostic stratification based on gene signature of IFN‐Mac_CXCL9. (B) Differentially expressed genes between IFN‐Mac_CXCL9 in tumours from the three cancer types and control tissues. (C) Bar plot showing enriched pathways of up‐regulated gene signatures of IFN‐Mac_CXCL9 in tumours from the three cancer types. (D) Kaplan–Meier curves for overall survival generated from TCGA cohorts of ovarian, endometrial and cervical cancer, demonstrating significant prognostic stratification based on gene signature of Angio‐Mac. (E) Bar plot showing enriched pathways of gene signature of Angio‐Mac. (F and G) Differentially expressed genes (F) and differentially activated pathways (G) between Angio‐Mac in tumours and control tissues. (H) UMAP plot with colour‐coding demonstrating regulatory activity (left) and gene expression (right) of NFKB1 in macro/mono. (I) Kaplan–Meier curves for overall survival were generated from TCGA cohorts of ovarian and cervical cancer, demonstrating significant prognostic stratification according to the expression of NFKB1. (J) Bar plot showing pathways regulated by NFKB1.

IFN‐primed macrophages, specifically IFN‐Mac_CXCL9, accumulated significantly in tumour samples, prompting further analysis. Analysis of TCGA datasets indicated that elevated IFN‐Mac_CXCL9 signature expression is associated with improved survival in patients with ovarian, endometrial and cervical cancers (Figure 3A). This trend was further validated by multivariate Cox regression controlling for age, stage and histological grade (Figure S16). Signature gene analysis of IFN‐Mac_CXCL9 revealed 108 significantly up‐regulated genes in tumour‐infiltrating cells including *HLA‐A*, *HLA‐DRB1* and interferon‐regulated genes (*CXCL9*, *CXCL10* and *IL4I1*) (Figures 3B and S7A and Table S4). Given that CXCL9/10 are chemokines involved in immunoregulation and inflammatory processes,^32^ and HLA‐related genes are essential for antigen presentation, we hypothesise that IFN‐Mac_CXCL9 plays a role in recruiting immune cells and presenting antigens within the TME. Functional enrichment analysis confirmed that tumour‐infiltrating IFN‐Mac_CXCL9 participates in MHC class II protein complex assembly and positively regulating lymphocyte activation (Figure 3C).

**FIGURE 4:**
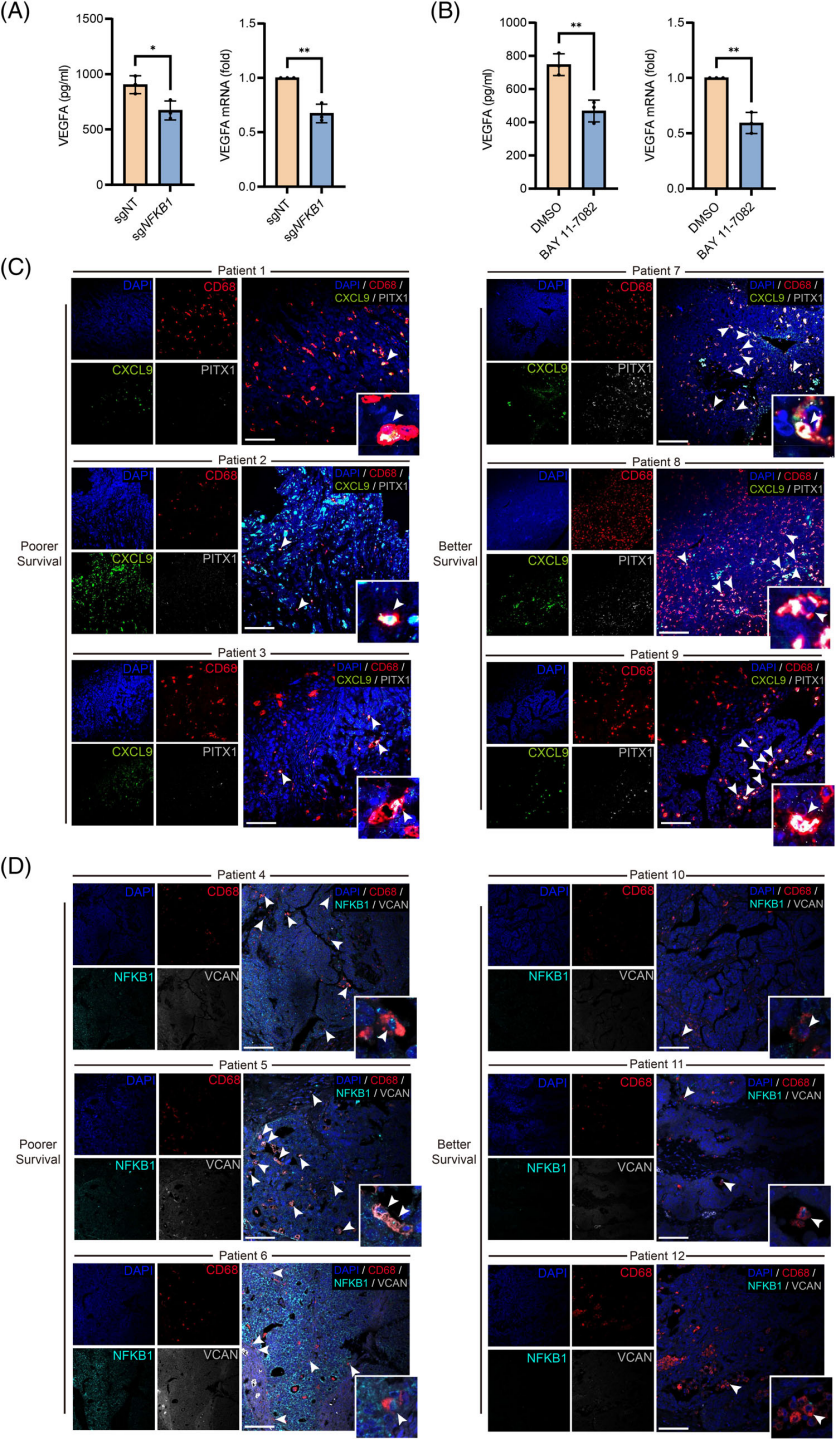
Validation of the regulatory function of NFKB1 and the prognostic relevance of IFN‐Mac_CXCL9 and Angio‐Mac. (A and B) Bar plots showing VEGFA secretion and expression in THP‐1 derived M2 macrophages after NFKB1 knockout (A) or treatment with an IKK inhibitor–BAY 11‐7082 (B). (C‐D) Representative images of mIHC staining for IFN‐Mac_CXCL9 (C) using CD68, CXCL9 and PITX1 and Angio‐Mac (D) using CD68, VCAN and NFKB1. The arrows indicate cells positive for all three markers. Scale bars, 100 µm.

Angio‐Mac has been reported in various cancer types^33^, ^34^, ^35^, ^36^, ^37^ and is known to accumulate in hypoxic regions,^38^ promoting tumour progression and metastasis. Survival analysis revealed significantly poorer outcomes in patients with higher levels of Angio‐Mac in TCGA OV, UCEC and CESC datasets (Figures 3D and S16). The consistency across multiple gynaecologic cancers prompted further investigation into this macrophage subset. Enrichment analysis of signature genes underscored Angio‐Mac's contribution to vasculature development, angiogenesis and participation in the NF‐κB signalling pathway and TNF response (Figure 3E). Differentially gene expression analysis revealed 39 up‐regulated genes in tumours, including *VEGFA*, *NFKBIA*, *S100A10* and *C15orf48* (Figures 3F and S7B and Table S5). These genes were involved in p53 signalling, hypoxia response and processes promoting cell adhesion and tumour cell invasion^39^, ^40^, ^41^ (Figure 3G). In contrast, 176 genes in non‐malignant samples were associated with translational initiation, wound healing and stress responses. As mentioned earlier, SCENIC analysis identified NFKB1 as the key TF for Angio‐Mac (Figures 2F and 3H). Survival analysis indicated that elevated *NFKB1* expression correlated with poorer survival outcomes in the TCGA‐OV and TCGA‐CSEC datasets (Figures 3I and S16). Enrichment of NFKB1 target genes indicated its role in TGF‐β signalling pathway, lipid storage, fibroblast migration and immune suppression through IL‐12 down‐regulation (Figure 3J and Table S6). *NFKB1* expression was largely restricted to monocytes and Angio‐Mac (Figure 3H), supporting the hypothesis that Angio‐Mac formation is driven by NF‐κB signalling in monocytes, leading to differentiation into a subset with angiogenic and immune suppressive properties. To validate the regulatory role of *NFKB1* and NF‐κB signalling in Angio‐Mac, we utilised THP‐1 cell line for experiments. Scoring on macrophages using classical M1 and M2 signatures^42^ showed that Angio‐Mac exhibited a strong M2‐like feature (Figure S7C). THP‐1 cells were differentiated into M0 macrophages using PMA and then polarised into M2 macrophages through IL‐4 and IL‐3 stimulation for subsequent experiments. *NFKB1* was knocked out using specifically designed sgRNA via a lentiviral CRISPR–Cas9 system (Figure S8A). The secretion of VEGFA in the supernatant from cell culture assessed by ELISA showed a significant reduction after *NFKB1* knockout, which was also confirmed by qPCR analysis of total RNA extracted from the cells (Figure 4A). Moreover, we delved into the function of NF‐κB signalling within Angio‐Mac, given its activation in these cells. M2 macrophages were exposed to the IKK inhibitor BAY 11‐7082, resulting in the suppression of the NF‐κB signalling pathway. Both ELISA and qPCR demonstrated a notable reduction in VEGFA levels following NF‐κB signalling inhibition (Figure 4B). The results strongly indicate that NF‐κB signalling, particularly the TF NFKB1, is crucial in the development of the Angio‐Mac subset.

To further validate the relationship between IFN‐Mac_CXCL9 or Angio‐Mac and clinical outcomes in patients, mIHC staining were performed on FFPE samples from 12 patients diagnosed with HGSOC, whom we followed up for 5 years after their initial surgery. Among the patients, six had a favourable outcome with no recurrence, whereas the remaining six experienced tumour recurrence and died during the follow‐up period (Table S3). IFN‐Mac_CXCL9 was defined as *CD68*+ macrophages co‐expressing *CXCL9* and its key TF, PITX1, while Angio‐Mac was defined as *CD68*+ macrophages co‐expressing *VCAN* and *NFKB1* based on its signature genes (Figure 2D). mIHC staining confirmed the presence of IFN‐Mac_CXCL9 and Angio‐Mac infiltration in tumours, indicated by the co‐localisation of their gene signatures. The presence of IFN‐Mac_CXCL9 was predominantly observed in patients with better survival outcomes (Figure 4C), whereas Angio‐Mac was more frequently detected in patients with poorer survival outcomes (Figure 4D).

### 3.4 Diversity of tumour‐infiltrating T cells

The central and complex roles of T cells in the TME have been widely recognised across various cancer types. As noted earlier, T cells are a predominant component in TIME of gynaecologic malignancies. To further explore this, we re‐clustered the T cells, with proliferating cells included for high *CD3D* expression (Figure 1D), after filtering genes in a manner similar to the approach used for macrophages/monocytes. Although *CD8A* expression was more pronounced compared with *CD4*, we found that the distribution of these two markers in T cells was almost mutually exclusive (Figure S9A). Consequently, T cells were categorised into three primary subsets: CD4 T cells, CD8 T cells and cycling T cells.

**FIGURE 5:**
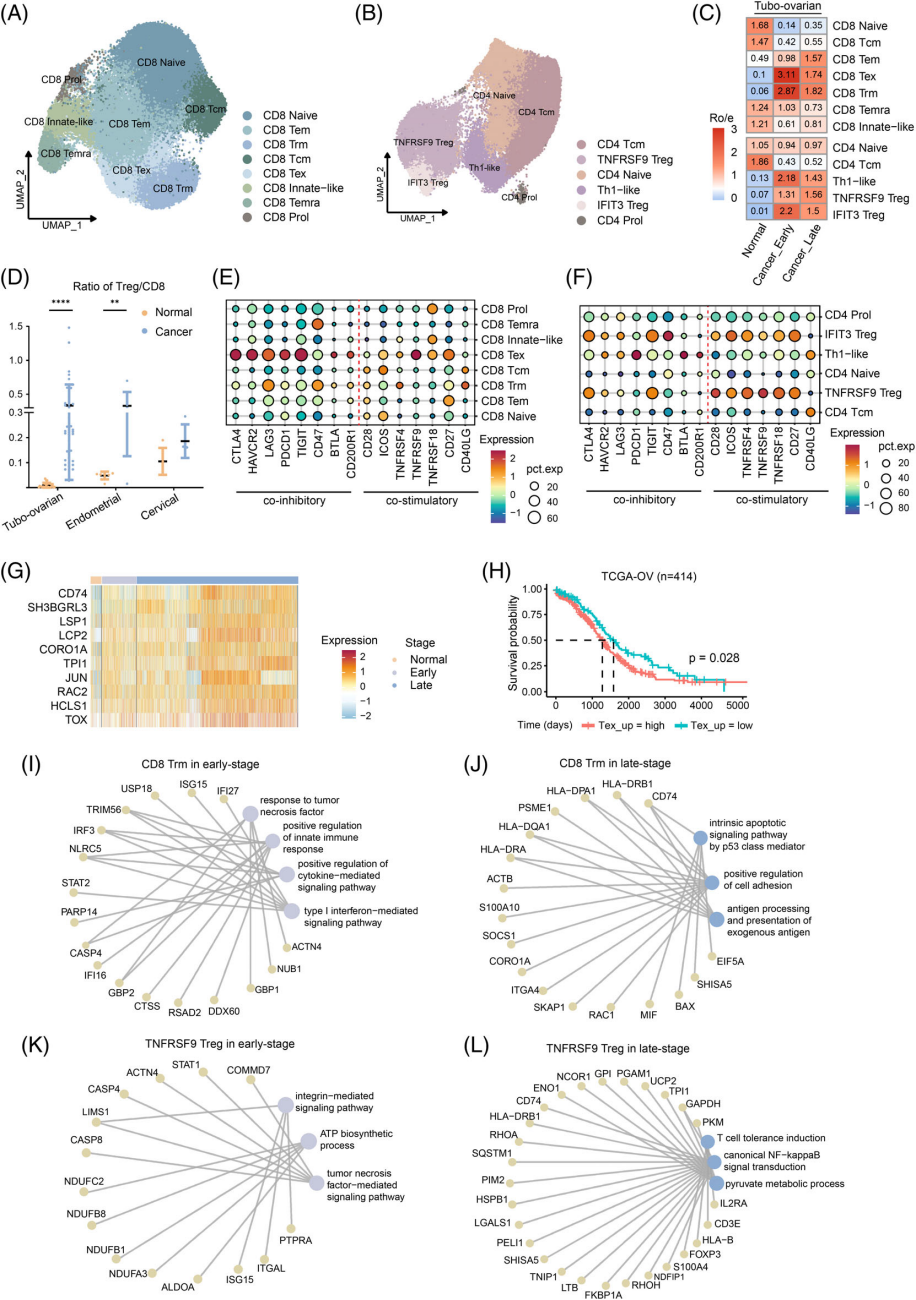
Dynamic roles of T cells in tumour progression. (A and B) UMAP plot displaying the subclusters of CD8 (A) and CD4 T cells (B). (C) Heatmap demonstrating the prevalence of each T cell cluster in control tissue and tubo‐ovarian tumours with different stages estimated by Ro/e score. (D) Fraction of Treg cells relative to the total CD8 T cell count in control tissues and tumours. The p values were calculated by Mann–Whitney U‐test. (E and F) Dot plot demonstrating the expression levels of co‐inhibitory and co‐stimulatory genes in various CD8 (E) and CD4 (F) T cells in gynaecological cancers. (G and H) Heatmap showing the expression of a specific gene set in CD8 Tex in different stages of tubo‐ovarian cancer (G) and Kaplan–Meier curves for overall survival demonstrating the prognostic stratification based on the expression of this gene set (H). (I and J) Typical up‐regulated genes and enriched pathways of CD8 Trm cells in early‐ (I) or late‐stage (J) tubo‐ovarian cancer. (K and L) Typical up‐regulated genes and enriched pathways of TNFRSF9 Treg cells in early‐ (K) or late‐stage (L) tubo‐ovarian cancer. ****p < 1.0e−4, **p < .01.

We subsequently analysed CD8 T cells, identifying eight subtypes (Figures 5A and S9B). CD8 T cell subsets were identified using established gene signatures from ovarian cancer research,^13^ including naive T cells (*GPR183*+), effector memory T cells (Tem; *GZMK*+), tissue‐resident T cells (Trm; *IFIT3*+), central memory T cells (Tcm; *DNAJB1*+), exhausted T cells (Tex, *CXCL13*+), TEMRAs (*CX3CR1*+) and proliferating T cells. A novel subset, termed innate‐like T cells, was identified, distinguished by cytotoxic markers different from traditional TEMRAs and marked by elevated FCER1G expression.^43^

We also performed subclustering on CD4 T cells, identifying six distinct subsets (Figures 5B and S9C). CD4 naive T cells expressed immature markers, including *CCR7*, *LEF1* and *TCF7*.^42^, ^44^, ^45^, ^46^, ^47^ CD4 central Tcm exhibited high expression of *ANXA1*,^20^, ^47^, ^48^ with moderate expression of naive markers, but also showed cytotoxic characteristics (*KLRB1*, *GZMA*), and were categorised by some studies as effector memory cells (TEM)^22^ or TEMRA.^44^ Th1‐like cells were identified by the expression of *CXCL13*, *BHLHE40* and *IFNG*, indicating their effector roles. Regulatory T (Treg) cells, known suppress immune effector cell activity and promote tumour immune escape,^49^, ^50^ were divided into two subgroups: one expressing *FOXP3* and *IL2RA* (also known as *CD25*) and marked by *TNFRSF9*,^51^ indicating activation, and the other showing *IFIT1* and *IFIT3*, suggesting responsiveness to interferons.

To better understand the relationships between these T cell subsets, we utilised Slingshot pseudospatial trajectory analysis (Figure S10B). CD8 naïve T cells could differentiate into either CD8 Tcm or Tem, with the former representing an immature status. The latter differentiated along three pathways, leading to Temra, Trm and Tex subsets. Similarly, CD4 naïve T cells could differentiate into CD4 Tcm, effective Th1‐like cells or immunosuppressive Treg cells.

### 3.5 Dynamics of T cell subsets in tumour progression

To investigate the function of various T cell subsets in cancer advancement, we compared the T cell composition in non‐malignant tissues and tumours at both early and late stages. In line with our prior research, Tex cells were predominantly enriched in tumours compared with non‐malignant tissues,^13^ with the most pronounced enrichment observed in early‐stage tubo‐ovarian cancer (Figure 5C and Table S7), suggesting continuous antigen stimulation of T cells by the tumour.^52^ A similar pattern was seen in Trm cells in tubo‐ovarian cancer. In contrast, naive and Tcm cells were primarily found in non‐malignant tubo‐ovarian tissue. The proportion of CD4 T cell subsets varied as cancer progressed. In tubo‐ovarian cancer, activated Treg cells (*TNFRSF9*+) tended to accumulate as tumour progressed from early to late stage, illustrating an immunosuppressive property. An elevated Treg/CD8 T cell ratio is linked to unfavourable outcomes in ovarian cancer.^53^, ^54^, ^55^ We calculated this ratio in our samples and found a general increase in all cancer types, though this change was not significant in cervical cancer (Figure 5D). This trend was particularly evident in tubo‐ovarian cancer, consistent with the significant accumulation of Treg cells discussed earlier.

The significant rise in Treg cells and CD8 Tex cells, accompanied by exhaustion markers, led us to examine these subsets more closely. Exhaustion markers such as *CTLA4*, *HAVCR2*, *LAG3*, *PDCD1*, *CD47*, *TIGIT*, *BTLA* and *CD200R1* are co‐inhibitory immune checkpoint receptors that prominently expressed in Treg cells and exhausted T cells,^50^, ^52^ facilitating the development of an immunosuppressive TIME and prompting immune evasion. These markers were enriched in Treg cells and CD8 Tex cells, as expected (Figure 5E, F). Specifically, Treg cells primarily expressed *CTLA4* and *TIGIT*, while CD8 Tex cells expressed all of the exhaustion markers, with the exception of *CD47*. Notably, CD8 Trm cells exhibited high levels of *LAG3* expression, consistent with our previous findings.^13^ Unlike the other exhaustion markers, *CD47* was predominantly expressed in CD8 Trm, Temra and IFIT3 Treg cells.

Costimulatory receptors are essential for T cell activation and sustaining T cell‐mediated immune responses,^56^, ^57^ alongside co‐inhibitory receptors. Our study identified an enrichment of CD28, ICOS and tumour necrosis factor receptor superfamily members, including TNFRSF4 (OX40), TNFRSF9 (4‐1BB), TNFRSF7 (CD27) and TNFRSF18 (GITR), in both Treg cells and CD8 Tex cells, indicating a dual role in immune regulation. Furthermore, *CD40LG*, a co‐stimulatory ligand that binds to *CD40* on antigen‐presenting cells (APCs),^57^ was highly expressed in CD8 Tcm, CD8 Trm, Th1‐like and CD4 Tcm cells, indicating their involvement in APC activation.^58^

To further investigate the molecular features of CD8 Tex, CD8 Trm and Treg cells at different stages of tumour progression, we performed differential gene analysis. We found that 10 genes within the CD8 Tex signature, including *CD74*, *SH3BGRL3*, *LSP1*, *LCP2*, *CORO1A*, *TPI1*, *JUN*, *RAC2*, *HCLS1* and *TOX*, were up‐regulated as tumours progressed from normal tissue to early‐ and late‐stage tubo‐ovarian cancer (Figure 5G). Elevated expression of this gene set correlated with poorer survival in ovarian cancer (Figure 5H). Functional enrichment analysis of genes highly expressed in early‐stage tumours indicated an up‐regulation of tumour necrosis factor signalling pathways in CD8 Trm and TNFRSF9 Treg cells (Figure 5I, K). Early‐stage Trm cells exhibited significant abundance in pathways linked to cytokine signalling and innate immunity modulation. In late‐stage tumours, genes highly expressed in Trm cells were involved in the intrinsic apoptotic signalling through the p53‐mediated pathway (Figure 5J). Conversely, genes up‐regulated in Treg cells were associated with T cell tolerance induction, NF‐κB signalling and pyruvate metabolic (Figure 5L), pathways linked to immune evasion and poor prognosis. In conclusion, T cells exhibit anti‐tumour activity during the early stages of cancer, while in the later stages, they may contribute to immune tolerance, facilitating tumour progression.

### 3.6 Characterisation of tumour‐infiltrating B cells

**FIGURE 6:**
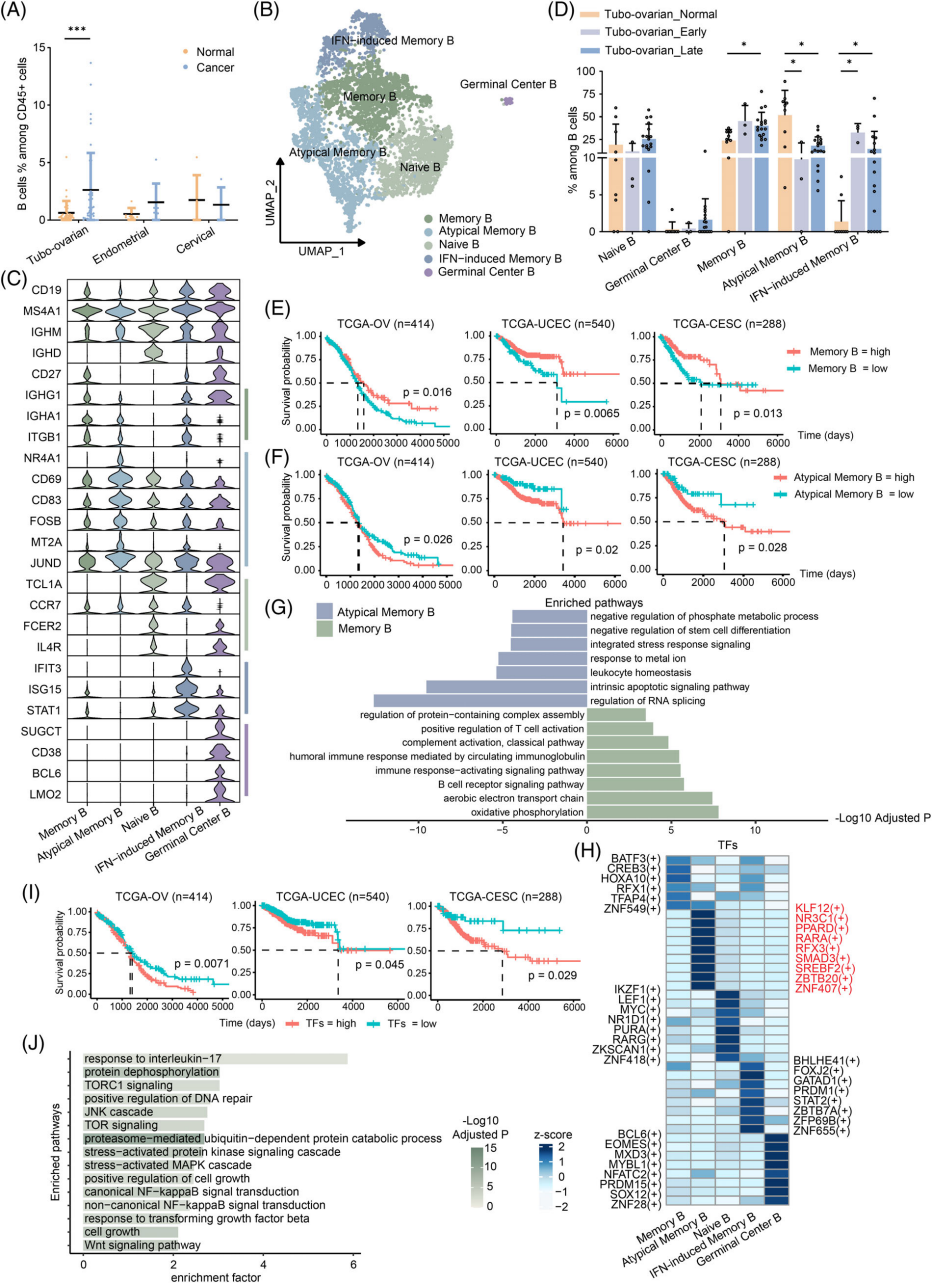
Identification and characteristics of B cells. (A) Percentages of B cells in tumours and control tissues. The p values were calculated by Mann–Whitney test. (B) UMAP plot displaying the re‐clustering B cells with distinct colours assigned to each subset. (C) Violin plot depicting the re‐clustering of B cells with distinct signature genes. (D) The fraction of each cluster relative to the total B cell count in control tissues and early‐stage or late‐stage tubo‐ovarian cancer. The p‐values were calculated by Kruskal–Wallis test. (E and F) Kaplan–Meier curves for overall survival generated from TCGA cohorts of ovarian, endometrial and cervical cancer, demonstrating significant prognostic stratification based on gene signature of memory B cells (E) and atypical memory B cells (F). (G) Bar plot showing differentially activated pathways in atypical memory B cells and memory B cells. (H) Heatmap showing specific TFs for each subset of B cells. The activity of TFs was represented by scaled AUCell score. (I) Kaplan–Meier curves for overall survival were generated from TCGA cohorts of ovarian, endometrial and cervical cancer, demonstrating significant prognostic stratification based on the expression of specific TFs for atypical memory B cells. (J) Bar plot showing pathways regulated by ZBTB20. ***p < 1.0e−3, *p < .05.

While earlier investigations on lymphocytes in the TME of gynaecologic cancers mainly concentrated on T cells, current studies highlight the important roles of B cells across human cancers. B cells, integral to humoral immunity, differentiate into antibody‐producing plasma cells, present antigens to T cells and are potential targets for immunotherapy.^23^, ^59^, ^60^ In our analysis, we calculated the percentage of B cells in various datasets and observed a low proportion in normal ovaries and endometrial tissues. The proportion of B cells was notably higher in tubo‐ovarian cancer samples than in non‐malignant ones (Figure 6A).

Four subsets of B cells have been identified in metastatic ovarian cancer: naive B cells, germinal centre B cells, plasma cells and plasmablasts.^61^ However, a more detailed characterisation of B cell subsets in gynaecologic malignancies has not been established, partly due to the relatively low number of B cells in these cancers. After excluding samples lacking B cell‐related genes, we performed subclustering on the remaining samples based on intersection of the sequenced genes. We identified five distinct clusters of *CD19*+ *MS4A1*+ B cells (Figure 6B). Annotation based on published studies categorised these clusters as: memory B cells (*CD27*+ *IGHM*+), IFN‐induced memory B cells (*CD27*+ *IGHM*+ *IFIT3*+), atypical memory B cells (*CD27*− *IGHD*− *IGHM*+, also referred to as double‐negative B cells or exhausted B memory cells^60^), naive B cells (*CD27*− *IGHM*+ *IGHD*+ *TCL1A*+) and germinal centre B cells (*SUGCT*+) (Figure 6C). While regulatory B (Breg) cells, expressing *GZMB* and *IL‐10*, has been reported in breast cancer,^62^, ^63^ they were not detected in our dataset, and thus were not yet defined in gynaecologic malignancies. Analysis of B cell proportions revealed that IFN‐induced memory B cells were more prevalent in the three types of tumours compared with their non‐malignant counterparts, with statistical significance observed only in ovarian cancer (Figures 6D and S11B,C). The proportion of memory B cells increased in late‐stage tubo‐ovarian cancer, while atypical memory B cells decreased in both early and late stages. However, this trend was not observed in endometrial or cervical cancers.

Previous research indicated that *CD27*− *IGHD*− atypical memory B cells contribute to an immunosuppressive microenvironment and are linked to poorer prognosis, while memory B cells correlate with enhanced therapeutic efficacy and extended survival.^60^ To explore the prognostic potential of these two B cell subsets in gynaecologic malignancies, we performed survival analysis. Interestingly, we found that elevated memory B cell signature expression correlated with improved outcomes, while atypical memory B cell signature was linked to poorer prognosis in the TCGA OV, UCEC and CESC datasets (Figures 6E, F and S16). Atypical memory B cells exhibited high expression of activation markers such as *CD83*, *NR4A1* and *CD69*,^60^, ^64^, ^65^, ^66^ along with stress‐related genes like *HSPH1* and *FOSB* (Figure 6C). Enrichment analysis revealed that atypical memory B cell signatures were linked to RNA silencing, splicing, stress response and the negative regulation of both phosphate metabolic processes and stem cell differentiation (Figure 6G). In contrast, memory B cells expressed high levels of *ITGB1*, *IGHA* and *IGHG* (Figure 6C), indicative of their role in promoting anti‐tumour immune response. Enrichment analysis further validated the participation of memory B cells in humoral immunity, T cell activation, complement activation and oxidative phosphorylation (Figure 6G).

We also investigated the TFs involved in B cell differentiation through SCENIC analysis. This analysis identified KLF12, NR3C1, PPARD, RARA, RFX3, SMAD3, SREBF2, ZBTB20 and ZNF407 as the top TFs for atypical memory B cells (Figure 6H), which we found to correlate with unfavourable prognosis (Figures 6I and S16). Among these TFs, ZBTB20 has been reported to increase the malignancy of various cancers.^67^ Our analysis identified that ZBTB20 modulates 1197 genes in B cells, influencing processes such as protein dephosphorylation, stress response, NF‐κB signalling and TOR signalling pathways, thereby reinforcing its pro‐tumour function (Figure 6J and Table S10). In contrast, many of the key TFs for memory B cells, including BATF3, CREB3, HOXA10, RFX1, TFAP4 and ZNF549, were also shared by IFN‐induced memory B cells, suggesting a similar evolutionary trajectory for these two subsets.

### 3.7 Crosstalk between cells within the TME

**FIGURE 7:**
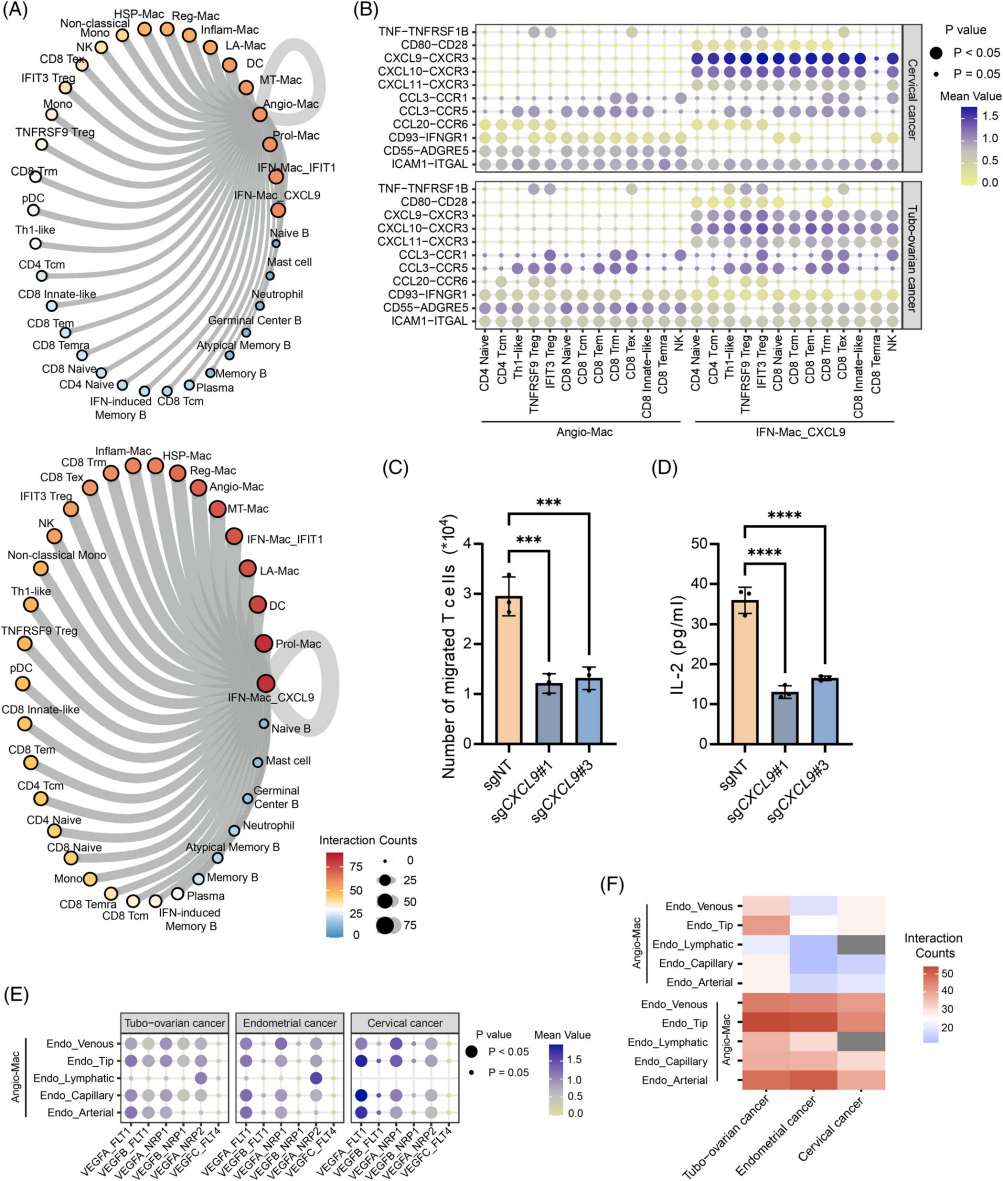
Communications between immune cells in the tumour microenvironment. (A) The communication network showing counts of significant interactions between Angio‐Mac or IFN‐Mac_CXCL9 (ligand) and the TIME (receptor). (B) Dot plots showing communication between Angio‐Mac, IFN‐Mac_CXCL9 (ligand) and T cells and NK cells (receptor) in tubo‐ovarian and cervical cancer. (C) Migration of Jurkat T cells estimated via flow cytometry upon cocultured with THP‐1 derived M1 macrophages in which NFKB1 has been knocked out. (D) Levels of IL‐2 in the supernatant of the Jurkat T–THP‐1 co‐culture system measured by ELISA. (E) Dot plots showing communication between Angio‐Mac (ligand) and endothelial cells (receptor) in tubo‐ovarian, endometrial and cervical cancer. (F) Heatmap displaying counts of significant interactions between Angio‐Mac and endothelial cells.

We applied CellPhoneDB to investigated cell–cell interactions within the TME of tubo‐ovarian, endometrial and cervical cancers. Significant interactions were observed between macrophage subtypes and between macrophages and T or NK cells, consistently across all three cancer types (Figure S12A–C). Given our findings that Angio‐Mac and IFN‐Mac_CXCL9 were correlated with worse and better survival, respectively, we focus on their communication with other immune cells. Overall, IFN‐Mac_CXCL9 exhibited more extensive communication within the TIME than Angio‐Mac, particularly with macrophages, T cells and NK cells, which aligns with its role in recruiting immune cells (Figure 7A). The crosstalk between Angio‐Mac and T/NK cells primarily occurred through CD55‐ADGRE5 and CD93‐IFNGR1 interactions (Figure 7B). In tubo‐ovarian cancer, interactions between IFN‐Mac_CXCL9 and T/NK cells were mediated by CXCL9/10/11–CXCR3 and CCL3–CCR5 interactions, with similar pattern observed in cervical cancer. Interestingly, the most significant interaction in tubo‐ovarian cancer was CXCL10–CXCR3, while in cervical cancer, it was CXCL9–CXCR3. This crosstalk was more moderate in endometrial cancer (Figure S12D). To further validate the recruitment of T cells by IFN‐Mac_CXCL9, chemotaxis assays were performed. Considering a highly M1‐like feature (Figure S7C), we generated THP‐1‐derived M1 macrophages as a resource for IFN‐Mac_CXCL9. THP‐1 differentiated M0 cells underwent M1 polarisation upon LPS and IFN‐γ stimulation. Following *CXCL9* knock out via sgRNAs (Figure S8B), M1 macrophages were co‐cultured with Jurkat T cells in a Transwell system for 4 h. As expected, the migration of T cells was significantly reduced after *CXCL9* knockout (Figure 7C). Additionally, we measured the concentration of IL‐2, a cytokine secreted by activated T cells, from the co‐culture system via ELISA. We found a significant decrease in IL‐2 secretion upon *CXCL9* knockout (Figure 7D), further supporting the functional recruitment of T cells through IFN‐Mac_CXCL9 via CXCL9. We also found that various types of T cells and NK cells secreted similar molecules that acted on Angio‐Mac or IFN‐Mac_CXCL9 (Figure S12E). Macrophage subsets were recruited by CD8 T cells and NK cells through the CCL5–CCR1 interaction, a mechanism not seen in CD4 T cells. In summary, our analysis of immune cell crosstalk enhances the understanding of macrophage–T cell interactions, potentially guiding the development of new immunotherapies to modulate the immune landscape in gynaecologic malignancies.

The angiogenic potential of Angio‐Mac has been discussed above; here, we further investigated its interaction with endothelial cells. Re‐clustering of endothelial cells identified five distinct subtypes: arterial, venous, capillary, lymphatic endothelial cells and tip cells^68^, ^69^, ^70^ (Figure S13A–C). CellphoneDB analysis verified that Angio‐Mac influences endothelial cells by secreting vascular endothelial growth factors (VEGF), notably highlighting the crucial role of VEGFA (Figure 7E). Moreover, Angio‐Mac primarily modulates lymphatic endothelial cells in tubo‐ovarian and endometrial cancers through the VEGFA–NRP2 axis. Additionally, all endothelial subsets were found to secrete APP, which acts on Angio‐Mac (Figure S13D). Notably, our analysis highlighted the Angio‐Mac–Tip cell interaction as the most prominent (Figure 7F), with tip cells recognised for their role in guiding vascular sprout.^68^, ^69^, ^70^, ^71^ In conclusion, the communications with endothelial cells provide further evidence of the pro‐angiogenic functions of Angio‐Mac.

## 4 DISCUSSION

The clinical response to immunotherapy in the three major gynaecological malignancies—ovarian, endometrial and cervical cancer—remain suboptimal, in part due to an incomplete understanding of the TIME. Recent advances in multi‐omics and single‐cell transcriptomic analysis have greatly enhances our grasp of the complex TIME, revealing innovative treatment approaches and targets.^72^, ^73^, ^74^ While many researchers have investigated the immune landscape of gynaecologic cancers, the majority have concentrated on individual tumour types or a restricted range of immune components.^11^, ^12^ This study presents a detailed single‐cell atlas of the immune landscape in gynaecological tumours, revealing novel insights into the dynamic shifts of immune cell populations throughout tumour progression. Analysis on immune cell profiles across non‐malignant tissues and tumours at different stages reveals crucial immune cell traits linked to clinical outcomes, potentially informing future therapeutic approaches.

One of the most notable findings were the significant increase in macrophage infiltration in tumours compared with normal tissues, supporting the established role of TAMs in promoting tumour progression, metastasis and immune evasion.^75^ We have identified two distinct populations of IFN‐primed macrophages, typically found in low quantities in normal tissues but significantly elevated in tumours, are pivotal in the immune response to cancer. In addition, using the TCGA database, we observed that gene signature of IFN‐Mac_CXCL9 was associated with better prognosis, likely due to their capability to recruit T cells and exert anti‐tumour effects. This finding aligns with our previous report identifying an M1‐like TAM subset with antitumour properties through *CXCL9/10/11* expression,^13^ and prior studies reporting that CXCL9/10/11‐producing macrophages and DCs enhance lymphocyte infiltration and sensitise tumours to immune checkpoint blockade.^76^ Analysis on cell–cell crosstalk and functional validation through chemotaxis assays provided direct mechanistic evidence that IFN‐Mac_CXCL9 could recruit and activate T cells. On the other hand, a subset with an angiogenesis‐associated gene signature, termed Angio‐Mac, was linked to poorer survival. Interestingly, this subset was also present in non‐malignant tissue, suggesting that its role may vary depending on the tissue context. In normal tissues, Angio‐Mac may contribute to stress responses and wound healing, while in tumours, it promotes cell adhesion and p53 signalling, both of which are associated with poor prognosis in gynaecological malignancies.^39^, ^41^ Furthermore, we identified NFKB1 as a key TF driving Angio‐Mac activation. NFKB1, a central player in the NF‐κB signalling pathway, has been pinpointed as a tumour‐promoting agent that fosters tumour cell growth and angiogenesis.^77^ The NFKB1‐driven gene signature includes pathways involved in IL‐12 down‐regulation, TGF‐β signalling, lipid storage and fibroblast migration. Notably, IL‐12, produced by APCs, is essential for T cell activation,^78^ whereas TGF‐β reduces the tumour‐fighting capabilities of macrophages and T cells and enhances Treg cell function.^79^ Our experiments confirmed that both genetic silencing of NFKB1 and pharmacological inhibition of IKK/NFκB signalling markedly reduce VEGFA expression in M2‐polarised macrophages, thereby validating NF‐κB as a key regulator of the Angio‐Mac subset. These findings elucidate the transcriptional mechanism of macrophage‐driven angiogenesis in gynaecologic cancers and highlight the therapeutic potential of targeting NF‐κB signalling to reduce tumour vascularisation and immune suppression. As anti‐VEGF therapy (e.g. bevacizumab) has been applied in the management of ovarian and cervical cancer, we advocate for research on whether NF‐κB pathway inhibitors could synergise with anti‐VEGF treatment to improve therapeutic efficacy. Overall, our findings regarding macrophage heterogeneity both align with and extend beyond established paradigms. The identification of two functionally discrete interferon‐primed macrophages within the same microenvironment, the demonstration of NFKB1 as a pro‐angiogenesis factor and the robustly consistent prognostic significance across all three cancer types offers novel insight into the TME of gynaecologic malignancies.

We also investigated the dynamics of T cell subsets during tumour progression. Our findings revealed a pronounced enrichment of CD8 Tex cells and Treg cells in the TME, compared with normal tissues. CD8 Tex cells exhibited high expression of multiple co‐inhibitory receptors, indicative of T cell dysfunction following persistent antigenic stimulation. Similarly, the up‐regulation of co‐inhibitory receptors on Treg cells has been well documented,^50^ and we observed that Treg cells in gynaecological malignancies predominantly expressed *CTLA4* and *TIGIT*. These findings reinforce the rationale for targeting immune checkpoints, such as CTLA4 and PD1, in order to reverse T cell dysfunction and improve clinical outcomes in these cancers. Interestingly, we also found that co‐stimulatory receptors were highly expressed on both CD8 Tex cells and Treg cells. This suggests that while these cells contribute to the immunosuppressive TME, they may also retain the potential for activation. Activating co‐stimulatory pathways may enhance T cell anti‐tumour responses, presenting a potential therapeutic intervention. In addition, we observed that CD8 Trm cells, which are critical for immune surveillance, also showed an increased presence in tumours, particularly in early‐stage ovarian cancer. Differential gene expression analysis indicated that both CD8 Trm cells and TNFRSF9 Treg cells contribute to immune responses in early‐stage tubo‐ovarian cancer. As tumours advance, these cells shift to roles that promote immune suppression and poorer clinical outcomes, underscoring the dynamic nature of T cell responses, which reveals a novel mechanism for the development of a suppressive TIME in advanced cancers. Immune checkpoint inhibitor therapies have demonstrated modest response in subsets of patients with cervical (e.g., Pembrolizuma^80^) and endometrial cancers (e.g., Dostarlima^81^), whereas ovarian cancer has remained largely refractory.^16^, ^17^, ^18^ Our analysis indicated that the abundance of immunosuppressive CD8 T cells and Treg cells, particularly in advanced tumours, along with reduced IFN‐Mac_CXCL9 activity, may contribute to therapeutic resistance. This finding offers a mechanistic basis for enhancing immunotherapy combinations by potentially activating IFN‐Mac_CXCL9 alongside immune checkpoint inhibitors.

Despite their smaller presence in the immune microenvironment, we identified five distinct B cell subsets in gynaecological tumours. Aligned with prior research, our findings indicated that memory and atypical memory B cells influence patient prognosis differently in gynaecological tumours.^60^ However, due to the limited number of B cells within these tumours, deeper analysis is required to thoroughly assess B‐cell‐directed therapeutic options.

While this study provides valuable insights, several limitations warrant recognition. First, the limited sample scope of endometrial and cervical cancer restricts the generalisability of our findings in these tumour types. Further studies with larger datasets are necessary to improve statistical power and refine the characterisation of immune heterogeneity in these cancers. Moreover, as our study integrated publicly available single‐cell datasets, inherent differences in tissue processing, sequencing depth and patient background may introduce potential biases. While we employed appropriate integration methods to minimise batch effects and sample heterogeneity, technical or biological variability between samples cannot be entirely exclude. Additionally, the translation of single‐cell‐derived gene signatures to bulk RNA‐seq data introduces interpretational challenge, partially due to variable immune cell proportions, which may confound the association between gene signatures and clinical outcomes. Another limitation pertains to the absence of Breg cells in our database. This could be due to their low abundance in gynaecological tumours or limitations in marker gene expression for Breg cell identification.

To summarise, this study provides a comprehensive single‐cell atlas of the immune landscape in gynaecological tumours, enhancing our comprehension of immune cell functional variety and their interconnections. Our research underscores the essential influence of macrophages and T lymphocytes in shaping the immunosuppressive TME, offering key insights for creating new therapeutic approaches. The identification of the Angio‐Mac subset, associated with poorer survival, along with the dynamic profiling of T cell subsets, offers new avenues for targeting immune checkpoints and co‐stimulatory pathways to improve clinical outcomes in gynaecological cancers.

## AUTHOR CONTRIBUTIONS

J.X. conceived the project. S.Y., S.L. and M.T. analysed the data and performed the experiment. S.Y. wrote the original draft. J.X. revised the paper. J.X. supervised the project.

## CONFLICT OF INTEREST STATEMENT

The authors declare no conflicts of interest.

## ETHICS STATEMENT

Patient tissue collection received approval from the Ethics Committee of Women's Hospital, School of Medicine, Zhejiang University (approval number: IRB‐20210064‐R).

## CONSENT

All authors have agreed to publish this manuscript.

## Supporting information



Supporting Information

Supporting Information

Supporting Information

## Data Availability

The datasets used and/or analysed during the current study are available from the corresponding author on reasonable request. Accession number for the public datasets used in our study was listed in Table S1, S2.
