# Supplementary material for: Single‐cell landscape of the tumour immune microenvironment in human gynaecologic malignancies
Source: Clin Transl Med. 2025 Nov 23;15(11):e70538. doi: 10.1002/ctm2.70538 (PMC12640613; doi:10.1002/ctm2.70538)
Supplement: Supplementary file 1 — Supporting Information [file CTM2-15-e70538-s001.pdf]

Figure S1-17

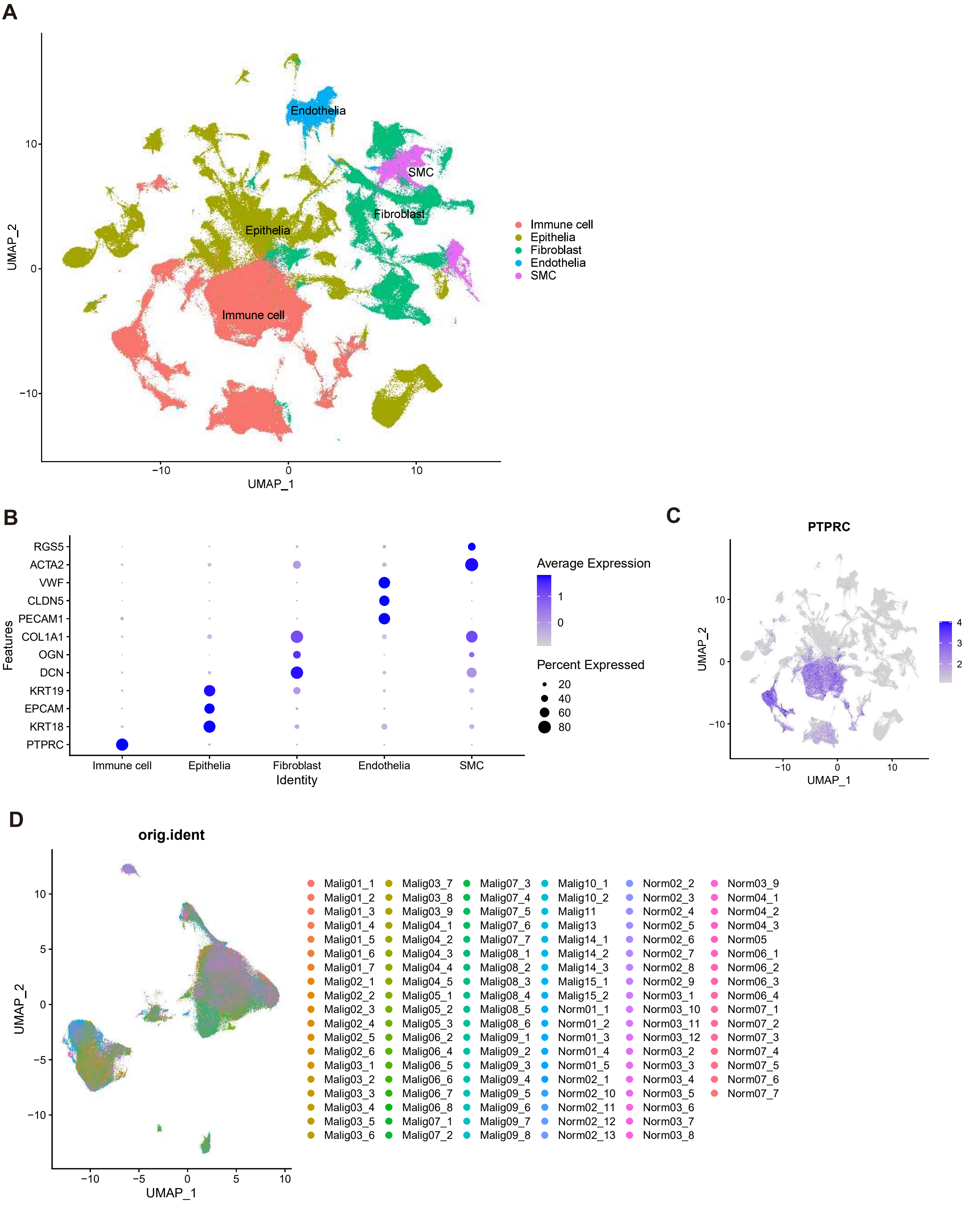

Figure S1. Identification of immune cells and their distribution across samples. (**A**) UMAP plot depicting main cell types in the integrated datasets. (**B**) Dot plots demonstrating the expression levels of specific marker genes in different cell types. (**C**) UMAP plot with color-coding demonstrating PTPRC (CD45) expression in all cells. (**D**) UMAP plot depicting distribution of immune cells across different samples.

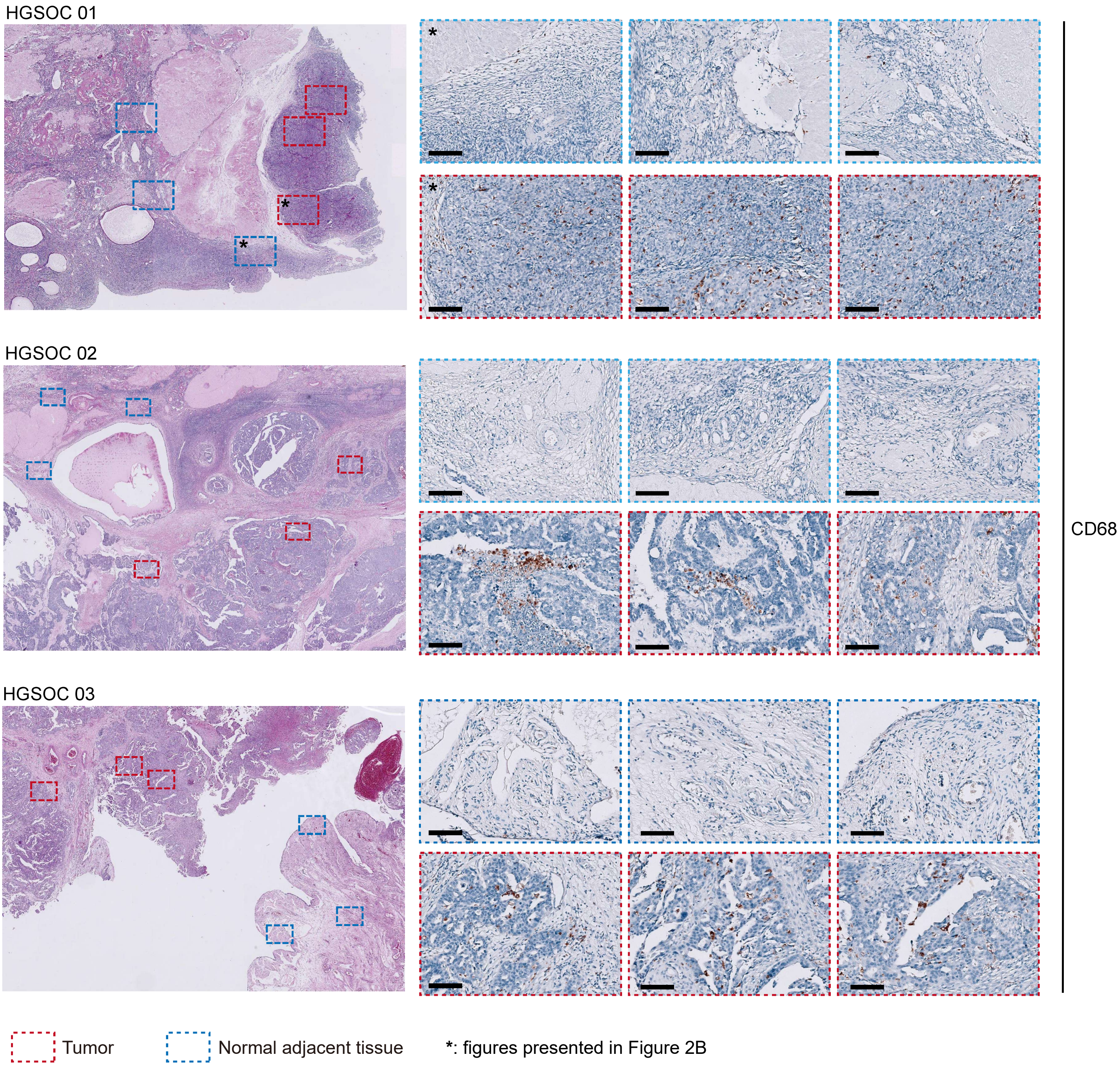

Figure S2. Representative images of H&E and IHC staining for CD68 in tumor and normal adjacent tissue in HGSOC. Scale bars, 100 μm.

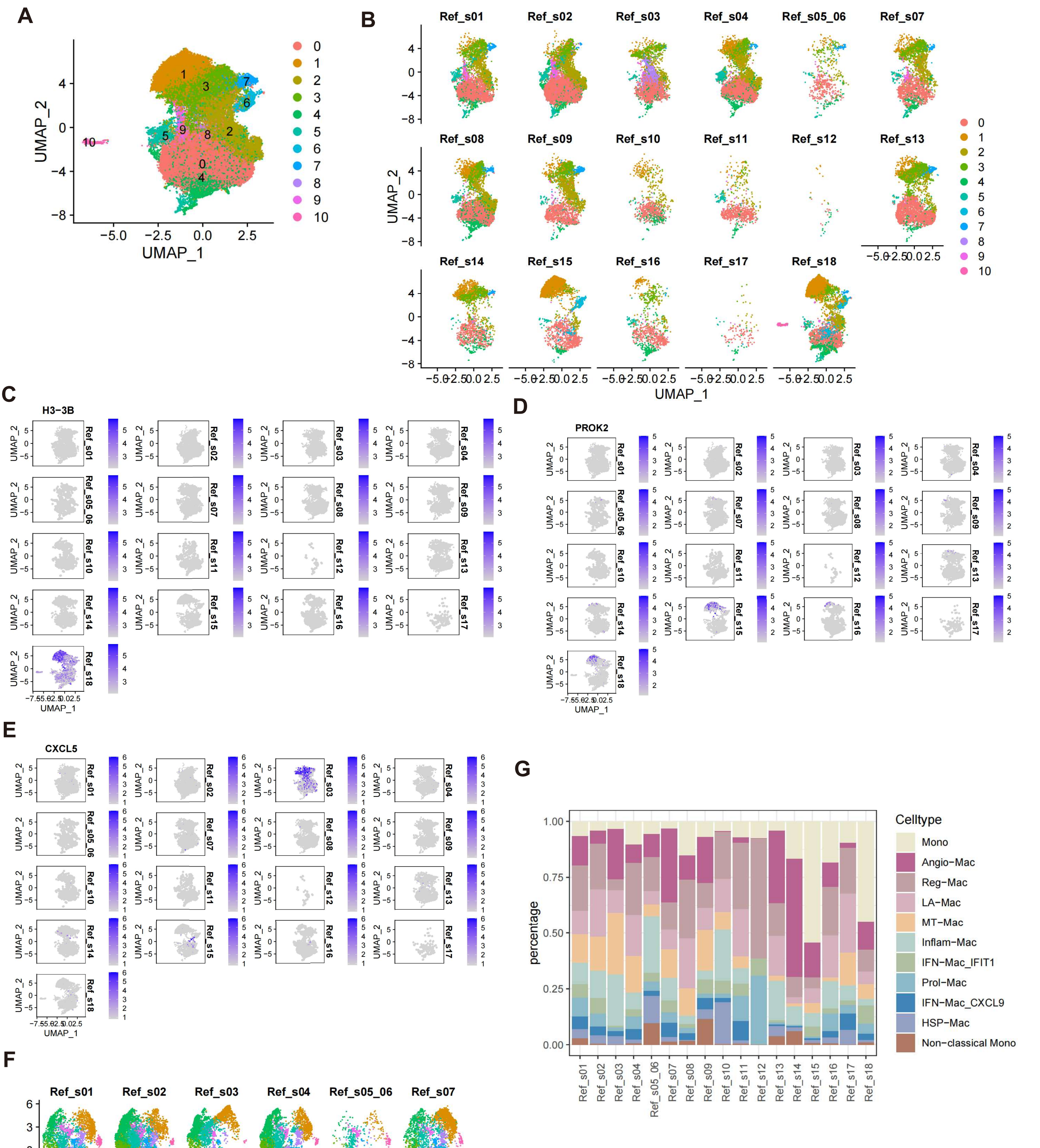

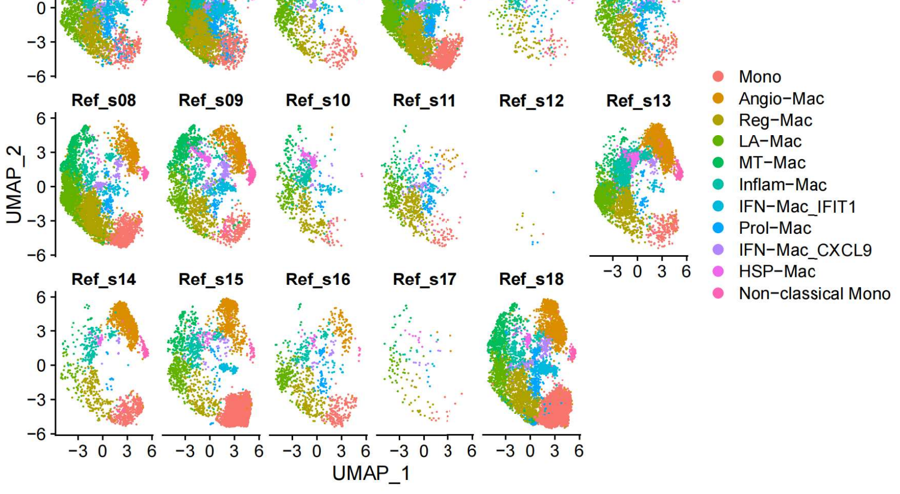

Figure S3. Disturbance of cohort-specific genes to the subclustering process. (**A**) UMAP plot depicting subclusters of the macrophages before filtering out cort-specific genes. (**B**) Distribution of the macrophage subclusters across different cohorts before filtering out cort-specific genes. (**C-E**) UMAP plot with color-coding demonstrating the expression of representative cort-specific genes in different cohorts. (**F-G**) UMAP plot and bar plot displaying the distribution of the macrophage subclusters across different cohorts after filtering out cort-specific genes.

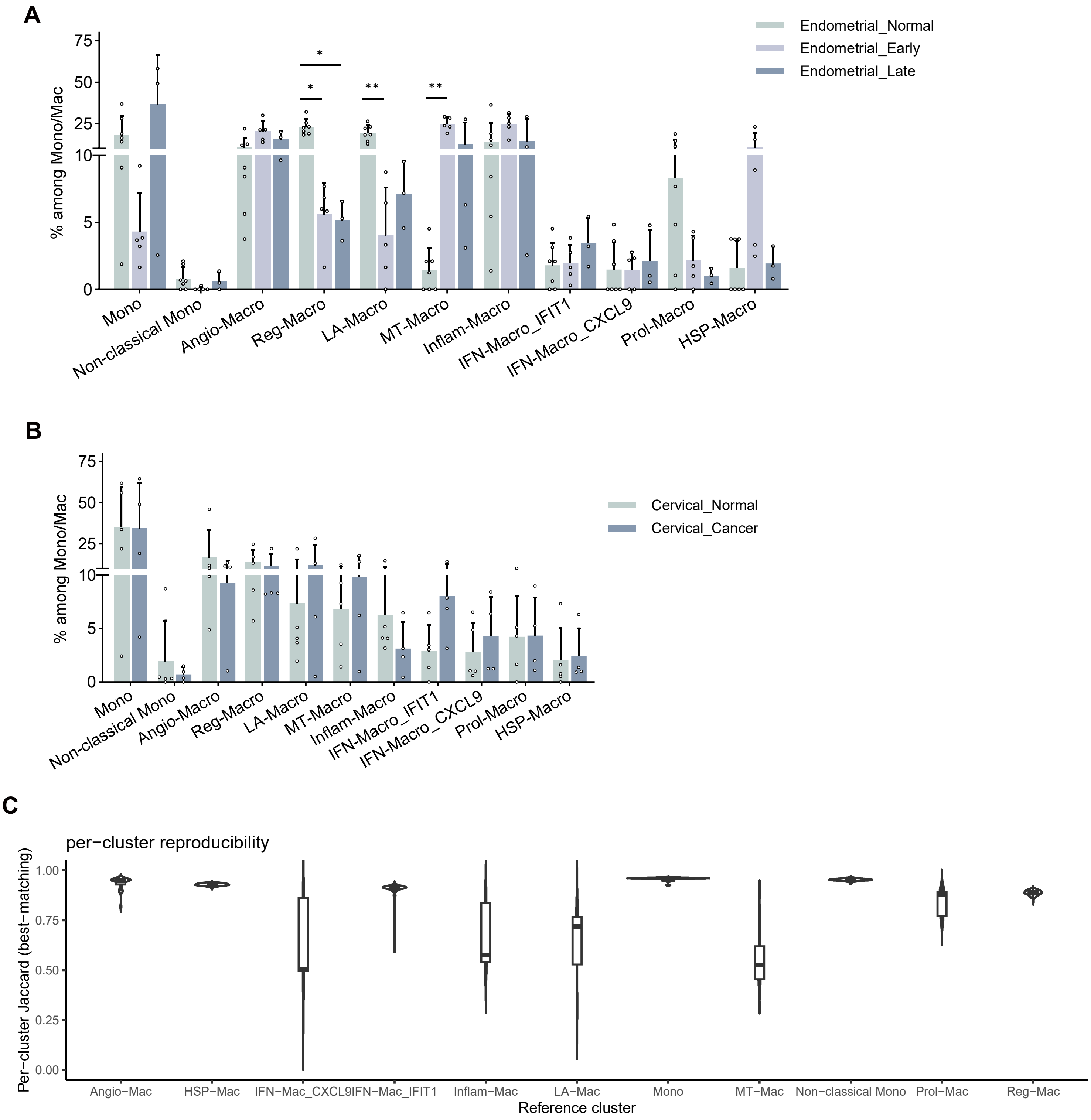

Figure S4. Characterization of Macro/Mono subsets. (**A**) Bar plot showing the fraction of each cluster relative to the total Macro/Mono count in control tissues and early-stage or late-stage endometrial cancer. The p values were calculated by Kruskal-Wallis test. (**B**) Bar plot showing the fraction of each cluster relative to the total Macro/Mono count in control tissues and cervical cancer. (**C**) Robustness and reproducibility of the macrophage subsets validated by subsampling-based robustness checks. **$p < 0.01$, *$p < 0.05$.

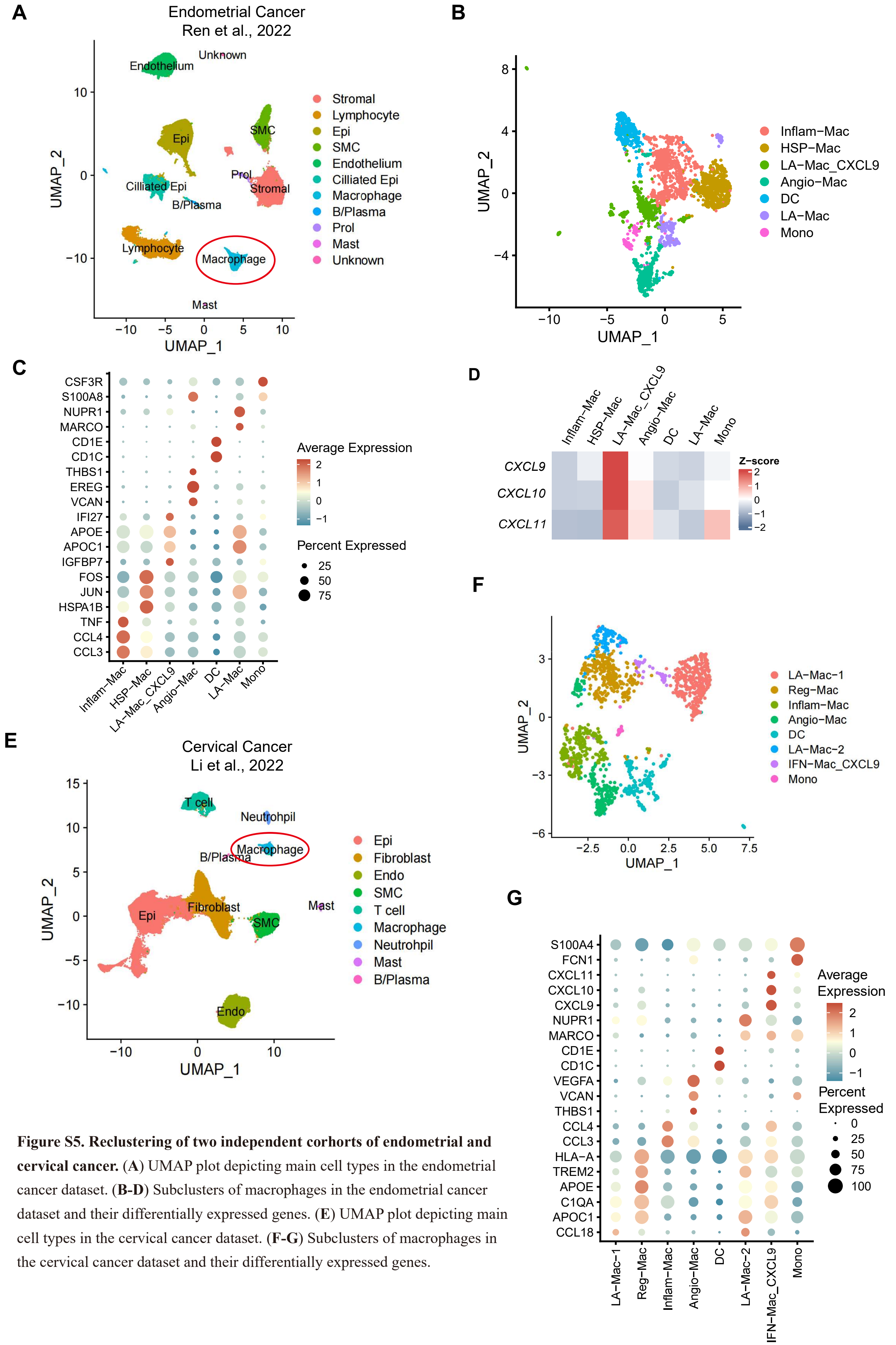

Figure S5. Reclustering of two independent corhorts of endometrial and cervical cancer. (**A**) UMAP plot depicting main cell types in the endometrial cancer dataset. (**B-D**) Subclusters of macrophages in the endometrial cancer dataset and their differentially expressed genes. (**E**) UMAP plot depicting main cell types in the cervical cancer dataset. (**F-G**) Subclusters of macrophages in the cervical cancer dataset and their differentially expressed genes.

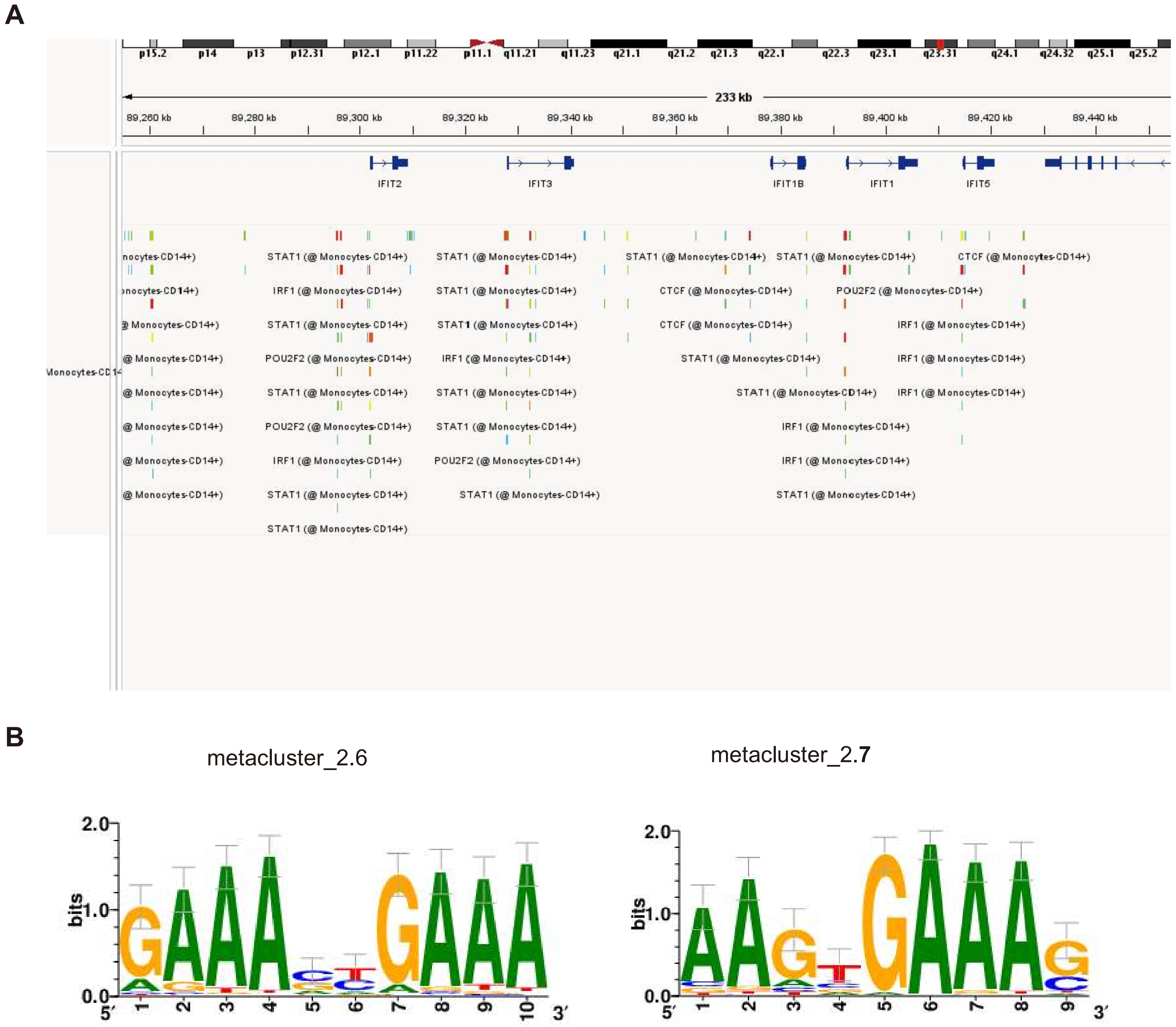

Figure S6. Validation of STAT1 as the key TF for IFN-Mac_IFIT1. (**A**) Binding of transcription factors, including STAT1, to key marker genes of IFN-Mac_IFIT1 demonstrated by multiple studies using Monocytes. (**B**) Identification of STAT1 binding motifs enriched by RcisTarget.

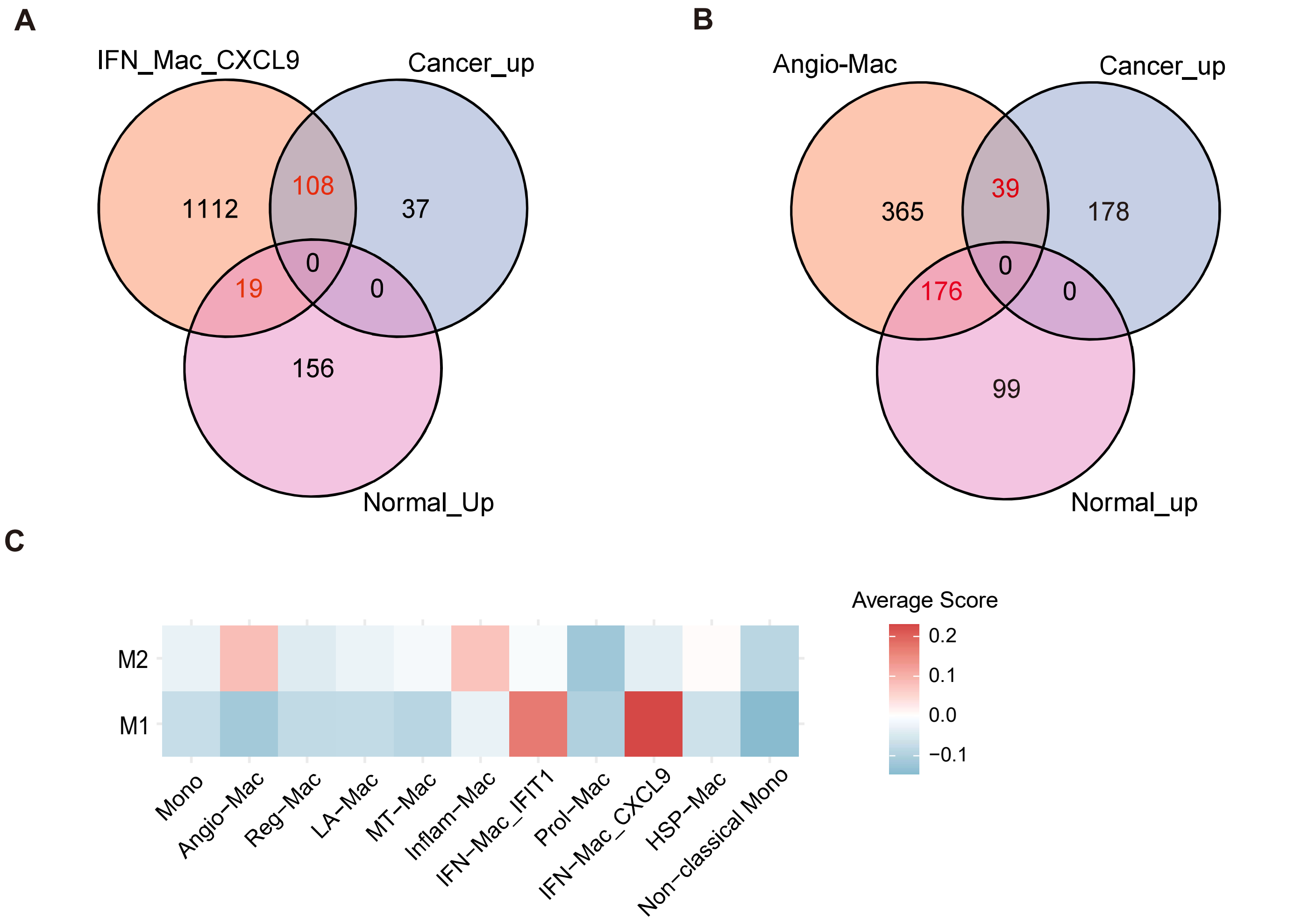

Figure S7. Characteristics of macrophage subsets. (**A-B**) Venn diagram showing intersection of IFN-Mac_CXCL9 (A) and Angio-Mac (B) signatures and genes upregulated in tumors or control tissues. (**C**) Heatmap depicting the gene module score for classical cell types M1 and M2 across the macrophage subclusters.

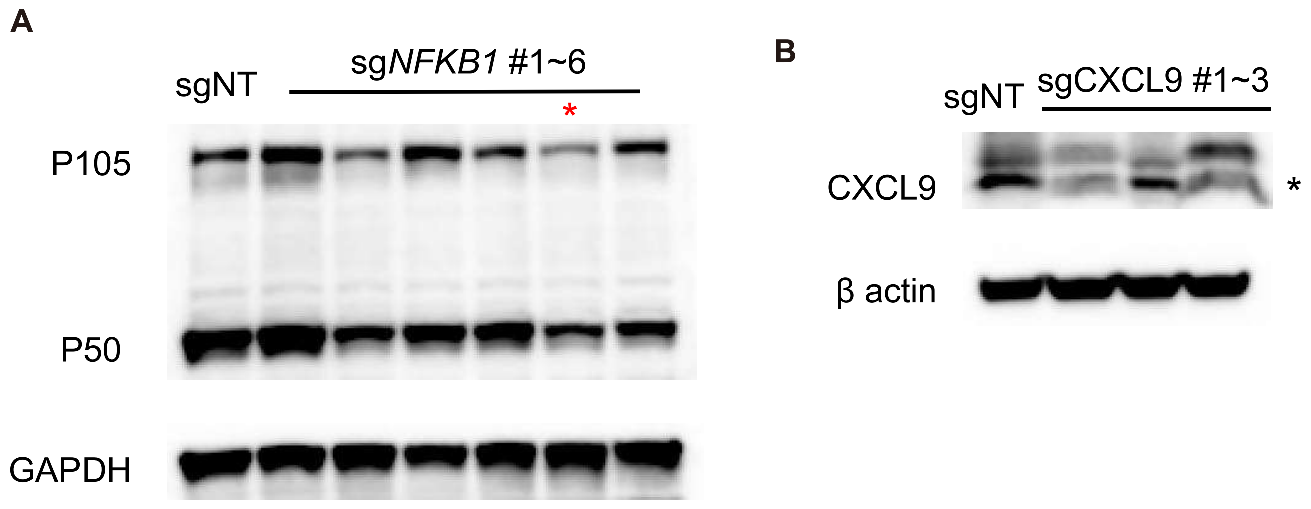

Figure S8. Confirmation of *NFKB1* and *CXCL9* knock out in the THP-1 cell line by western blot. sg*NFKB1* #5, sg*CXCL9* #1 and #3 were utilized for further experiments.

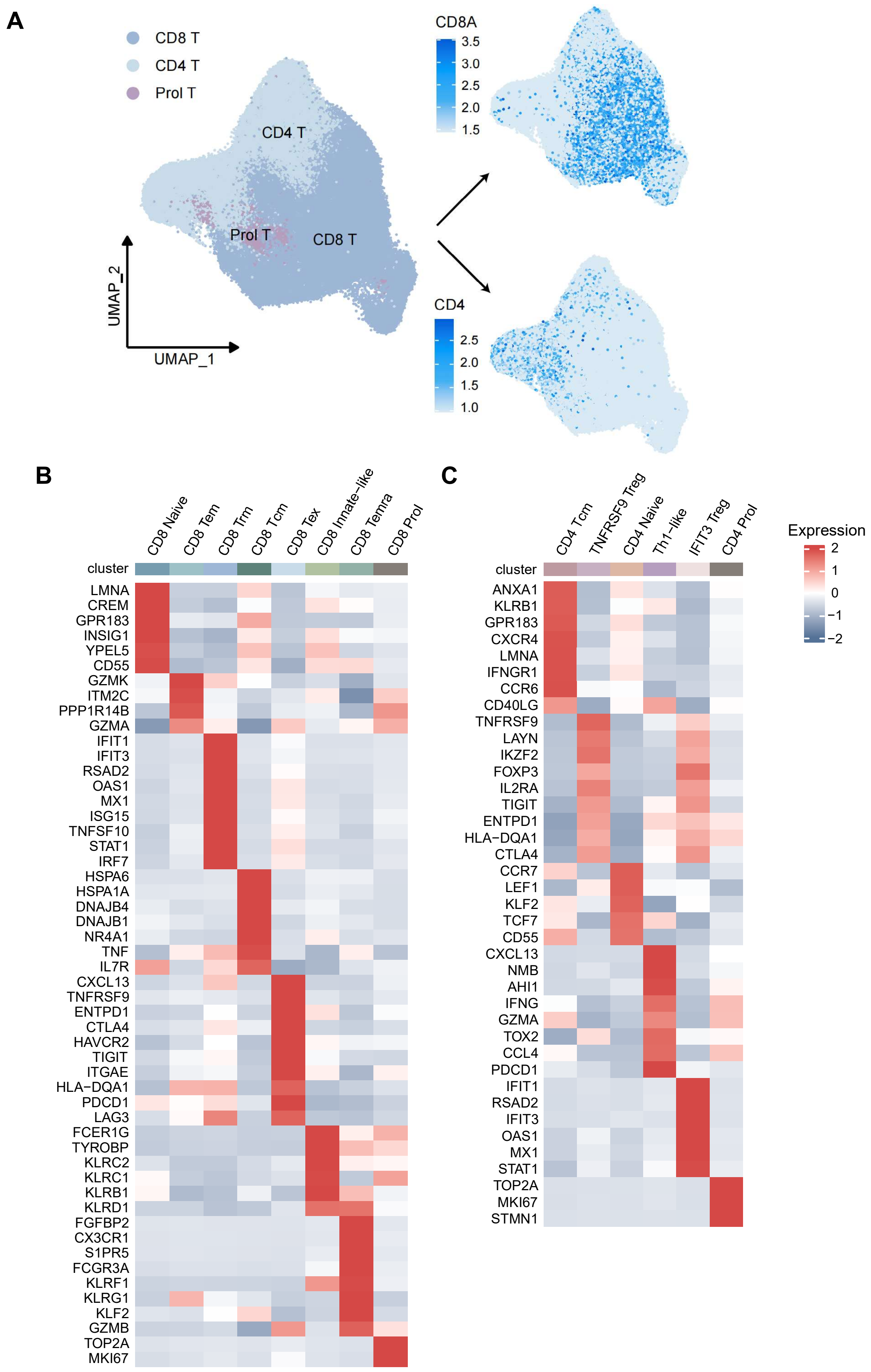

Figure S9. Identification of distinct T cell subsets. (**A**) UMAP plot displaying the various types of T cells (left) and expression of CD8A/CD4 (right). (**B-C**) Heatmap depicting the reclustering of CD8 T cells (B) and CD4 T cells (C) with distinct signature genes.

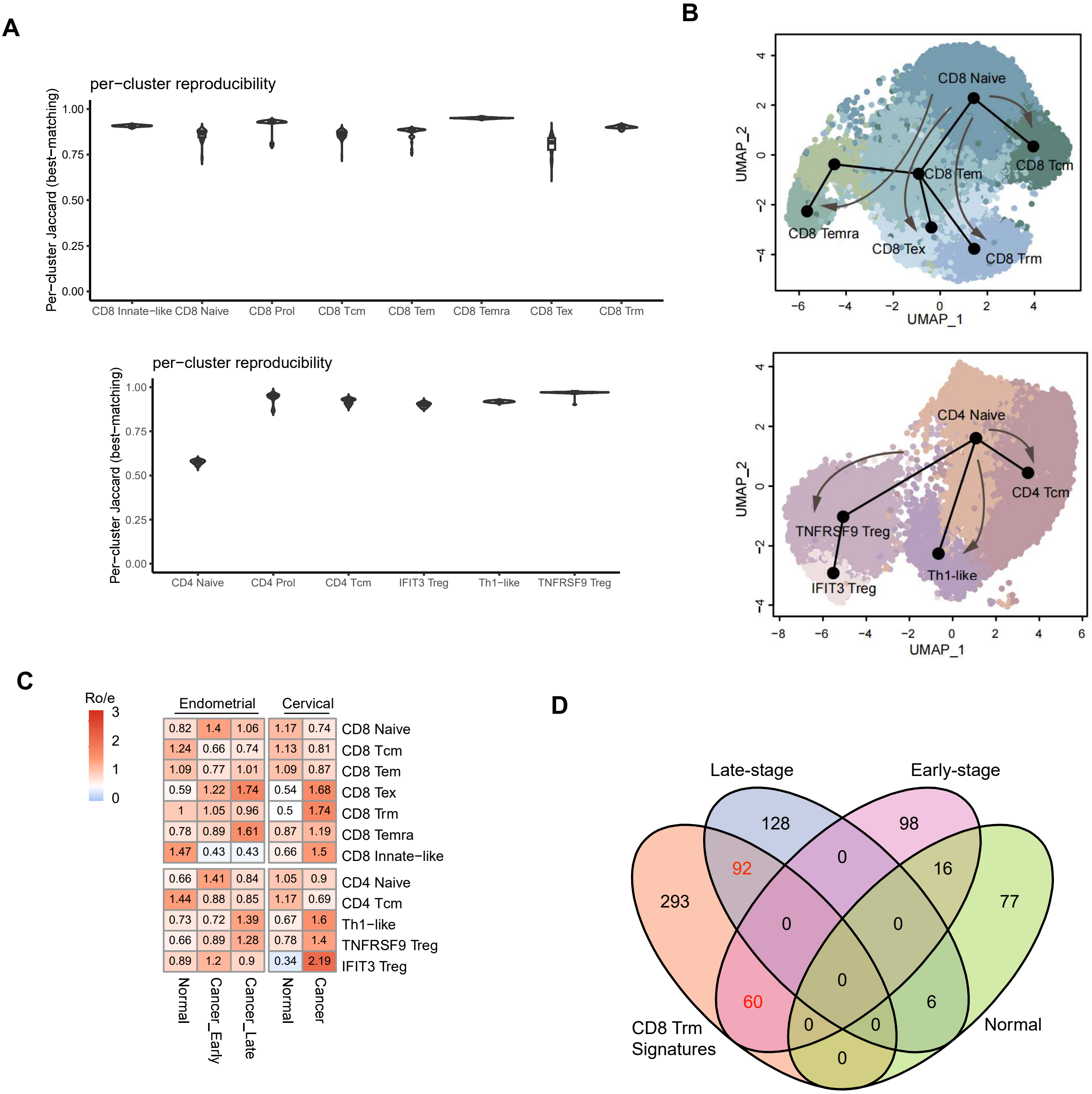

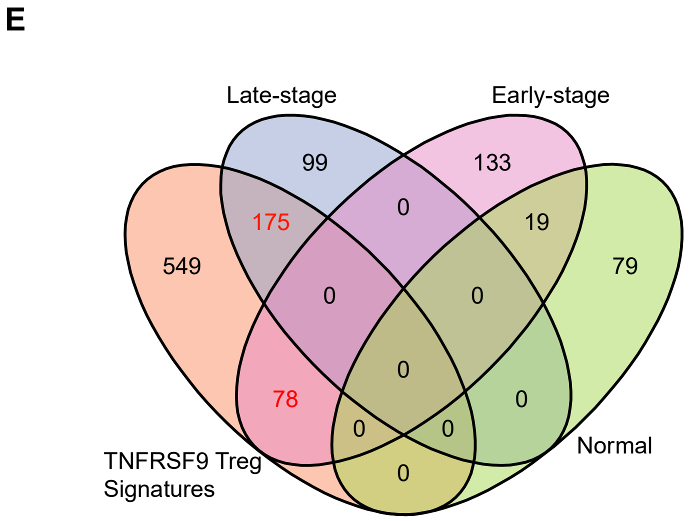

Figure S10. Characteristics of T cell subclusters. (**A**) Robustness and reproducibility of the Tcell subsets validated by subsampling-based robustness checks. (**B**) Slingshot pseudospatial trajectory analysis of CD8 and CD4 T cell subclusters. (**C**) Heatmap demonstrating the prevalence of each T cell cluster in control tissue and endometrial or cervical tumors with different stages estimated by Ro/e score. (**D-E**) Venn diagrams showing intersection of CD8 Trm (D) and TNFRSF9 Treg (E) signatures and genes upregulated in early-stage or late-stage tubo-ovarian cancers and control tissues.

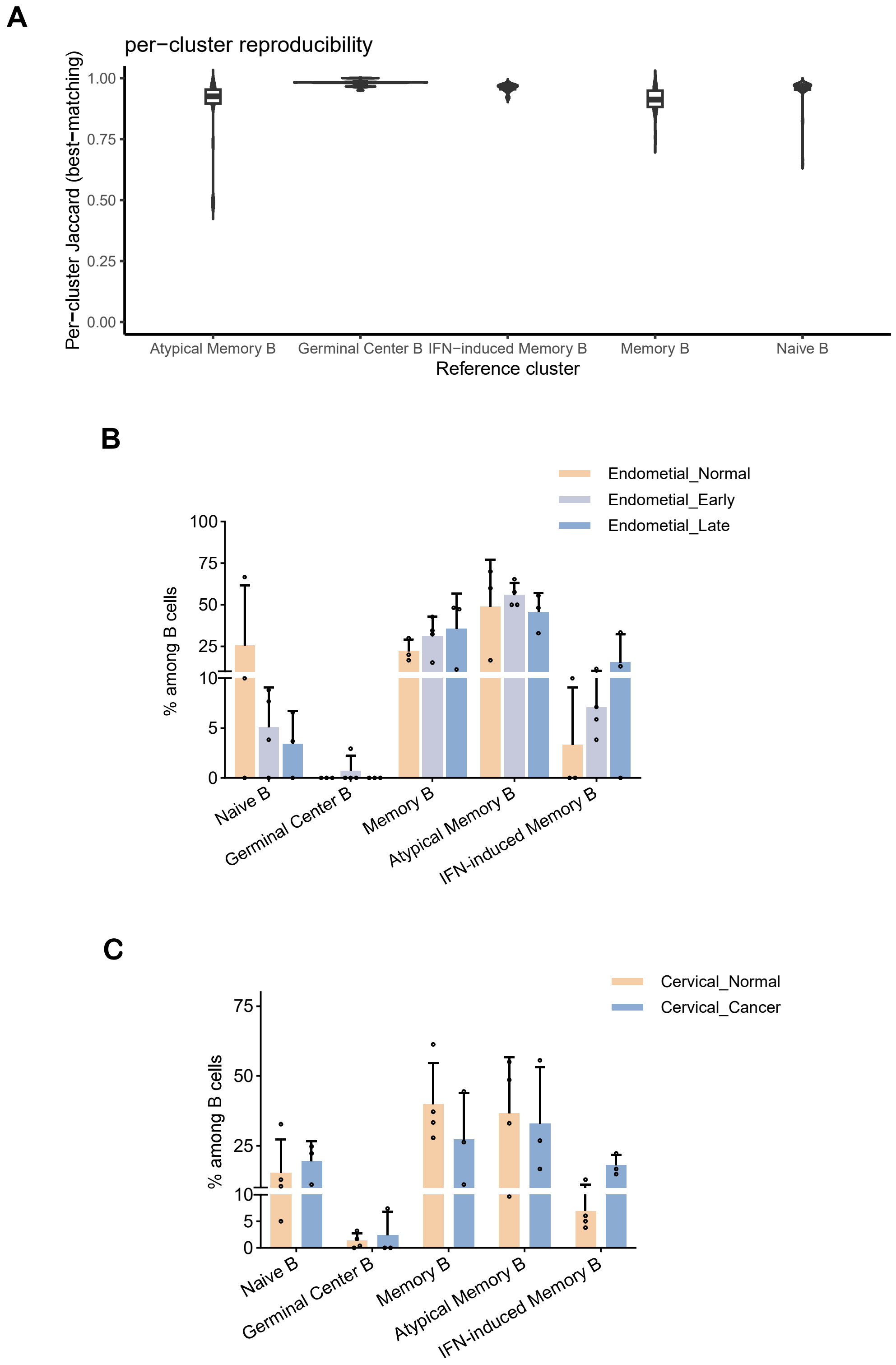

Figure S11. Reclustering of B cells. (**A**) Robustness and reproducibility of the B cell subsets validated by subsampling-based robustness checks. (**B-C**) Bar plots showing the fraction of each cluster relative to the total B cell count in control tissues and various stages of endometrial cancer and cervical cancer. The p-values were calculated by Kruskal-Wallis test (B) or Mann-Whitney test (C).

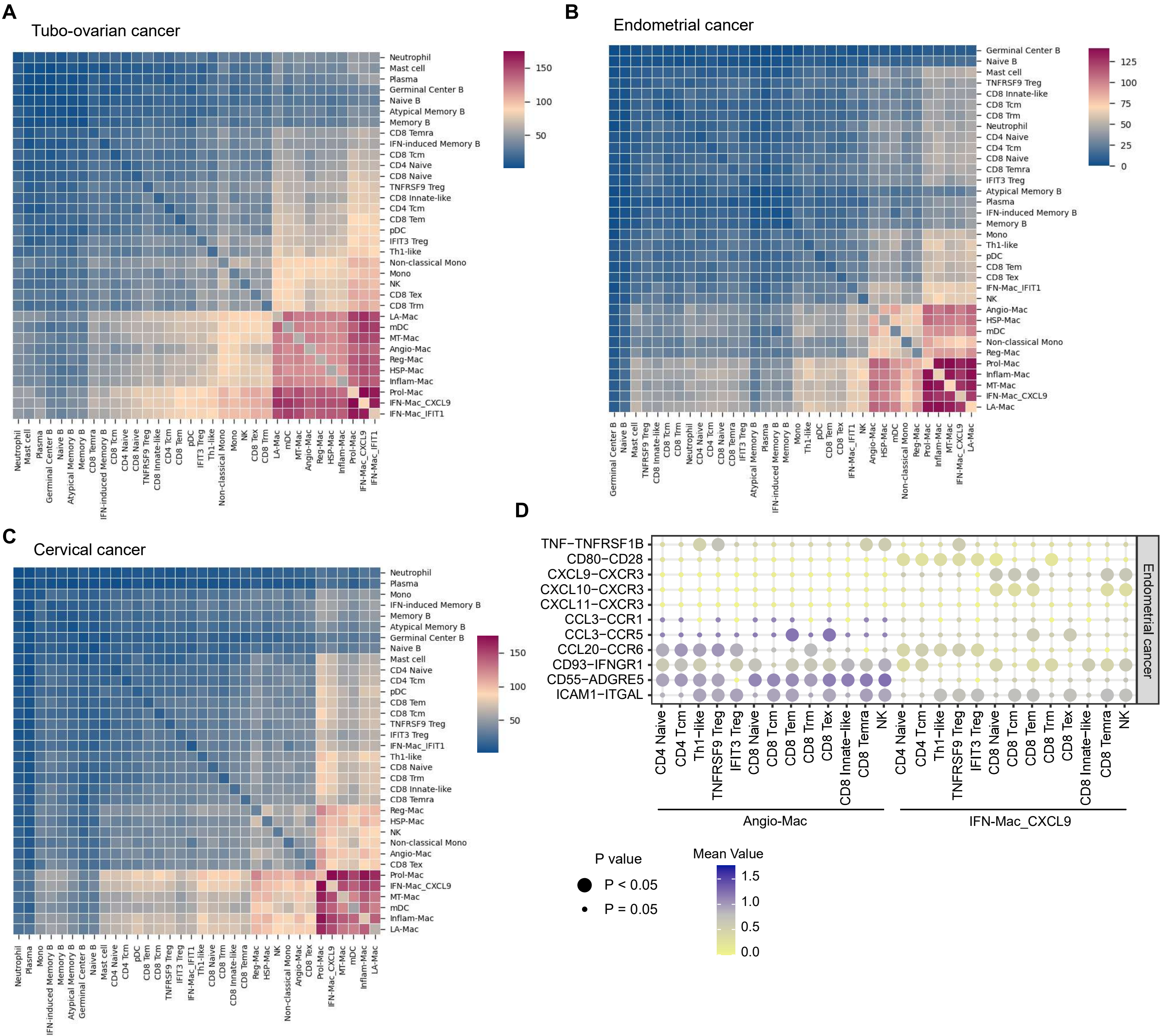

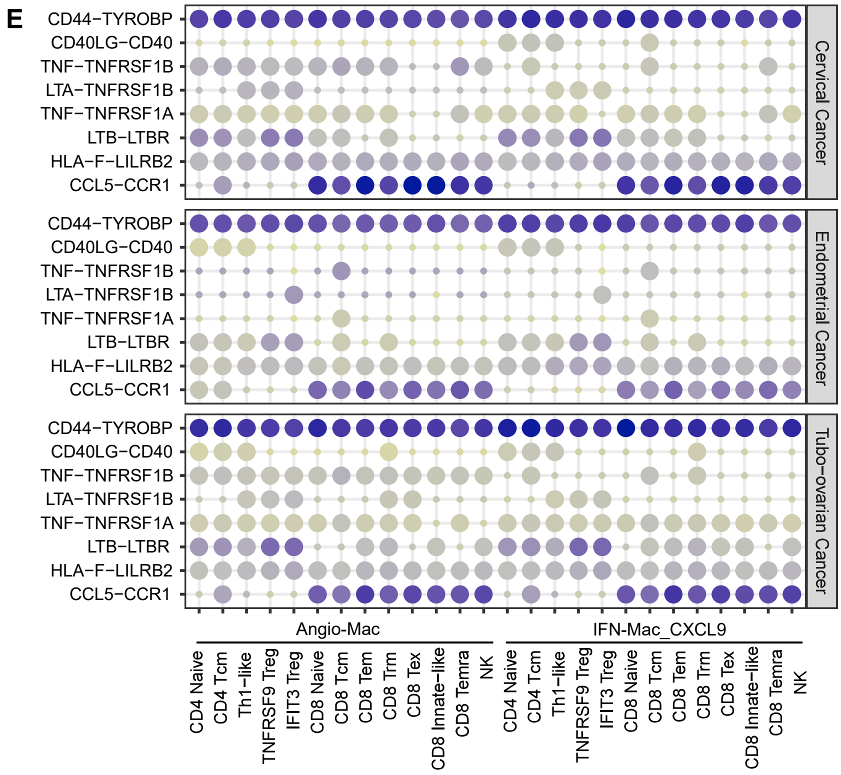

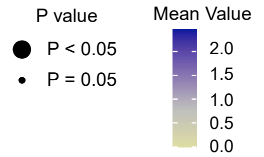

Figure S12. Cell-cell interactions in the tumor micro-environment. (**A-C**) Heatmap depicting significant interaction counts between cells in Tubo-ovarian cancer, endometrial cancer and cervical cancer. (**D**) Dot plot showing communication between Angio-Mac, IFN-Mac_CXCL9 (ligand) and T cells and NK cells (receptor) in endometrial cancer. (**E**) Dot plots showing communication between endothelial cells (ligand) and Angio-Mac (receptor) in tubo-ovarian, endometrial and cervical cancer.

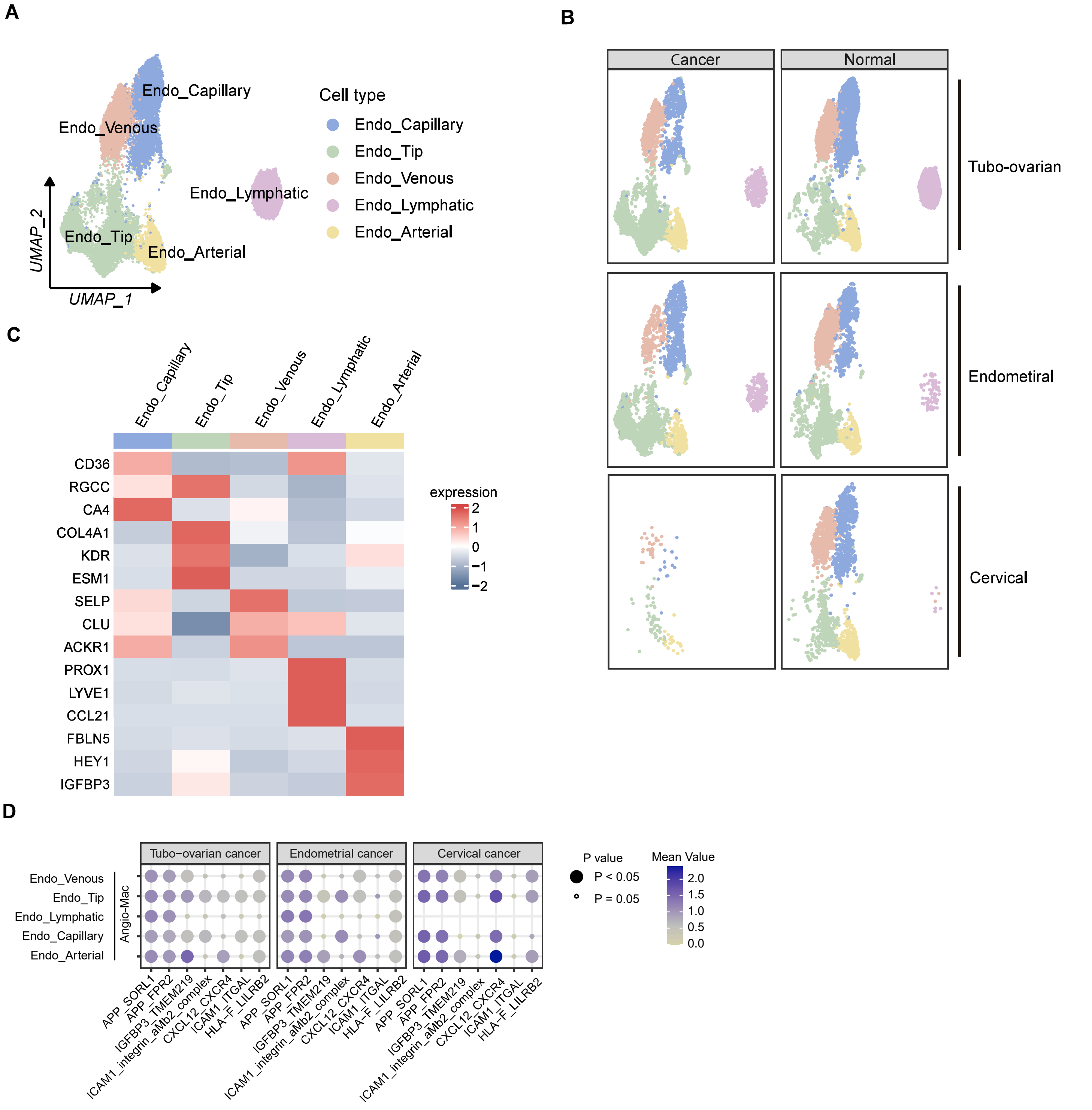

Figure S13. Features of endothelial cells in gynecologic malignancies. (**A**) UMAP plot depicting subtypes of endothelial cells in tumors and control tissues with distinct colors assigned to each cell type. (**B**) UMAP plot depicting subtypes of endothelial cells in tumors of tubo-ovarian cancer, endometrial cancer, cervical cancer and their nonmalignant counterparts. (**C**) Heatmap depicting the reclustering of endothelial cells with distinct signature genes. (**D**) Dot plots showing communication between endothelial cells (ligand) and Angio-Mac (receptor) in tubo-ovarian, endometrial and cervical cancer.

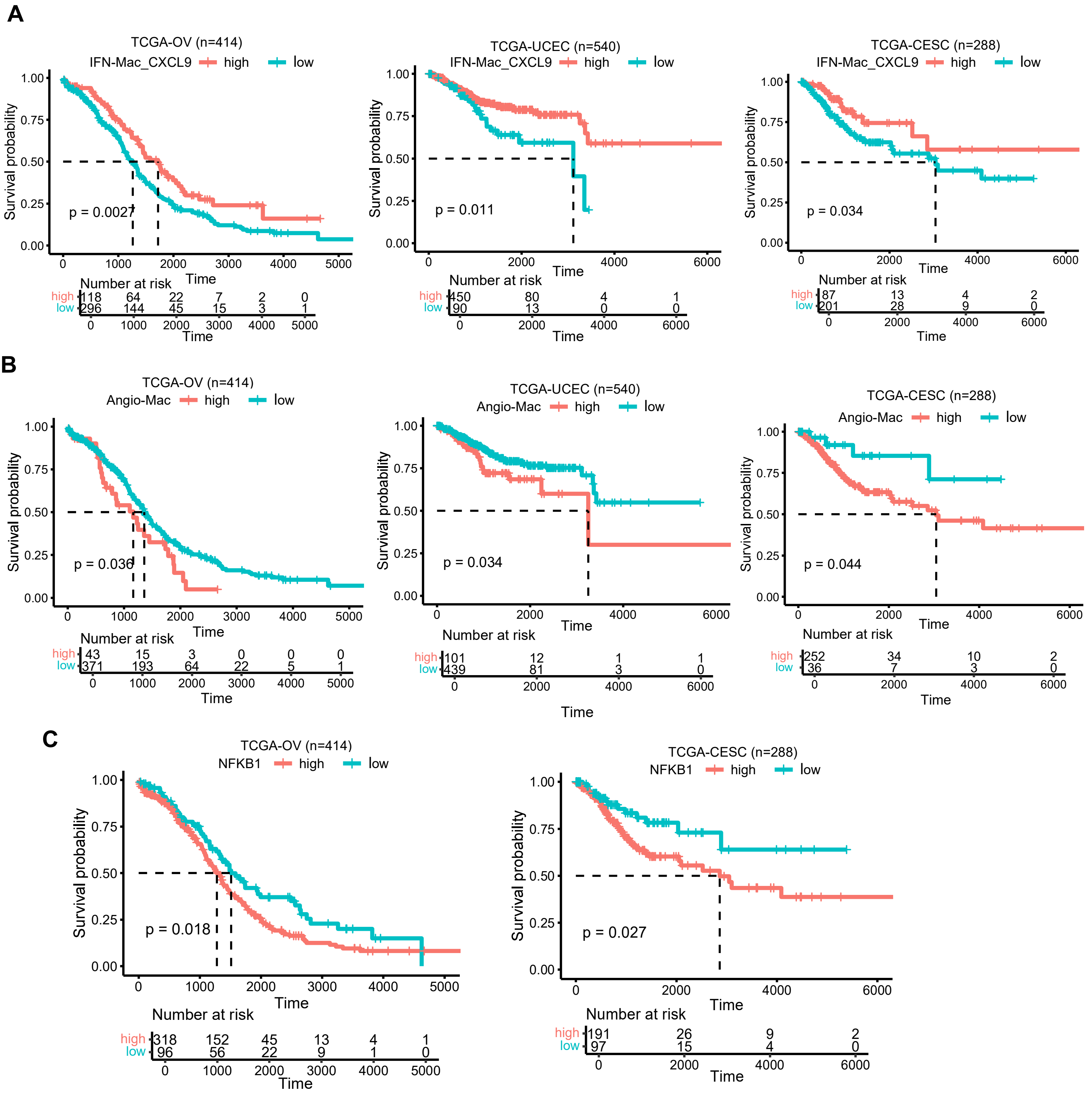

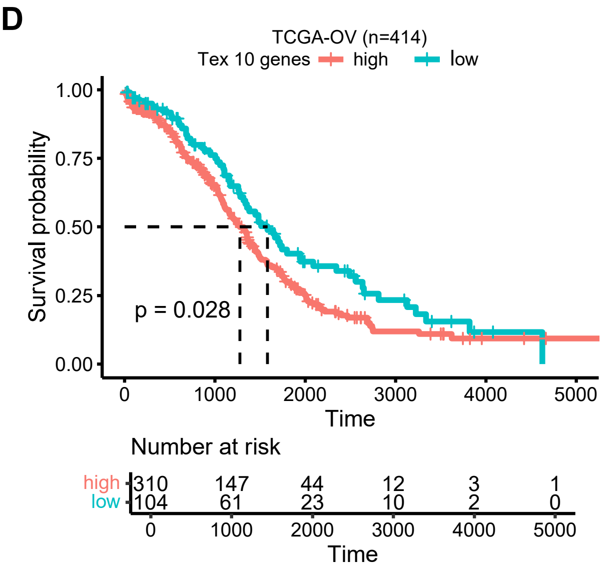

Figure S14. Kaplan-Meier curves with risk tables for overall survival generated from TCGA cohorts of ovarian, endometrial and cervical cancer, demonstrating significant prognostic stratification based on gene signature of IFN-Mac_CXCL9(A), Angio-Mac (B), the expression of NFKB1 (C) and a specific set of 10 genes within CD8 Tex signature (D).

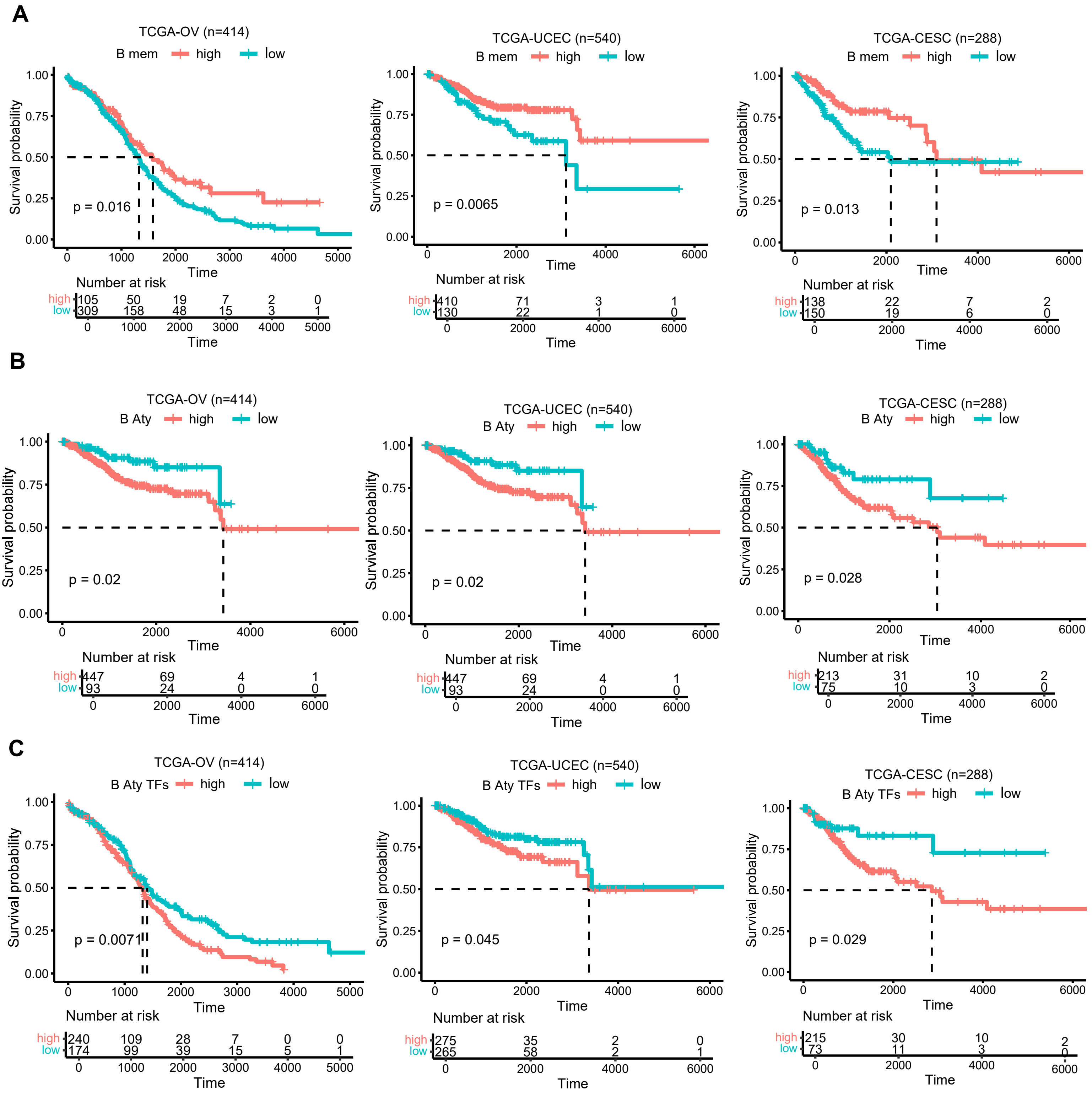

Figure S15. Kaplan-Meier curves with risk tables for overall survival generated from TCGA cohorts of ovarian, endometrial and cervical cancer, demonstrating significant prognostic stratification based on gene signature of memory B cells (A), atypical memory B cells (B) and major transcription factors for atypical memory B cells (C).

A

Multivariate Cox __TCGA-OV

Cell Population	HR (95% CI)	p−value
Angio−Mac	1.36 (0.91−2.05)	0.13678
IFN−Mac_CXCL9	0.64 (0.47−0.86)	0.00332
NFKB1	1.64 (1.21−2.22)	0.00160
Memory B	0.69 (0.50−0.94)	0.01822
Atypical memory B	1.38 (1.07−1.77)	0.01193
TFs for Atypical memory B	1.48 (1.15−1.91)	0.00268

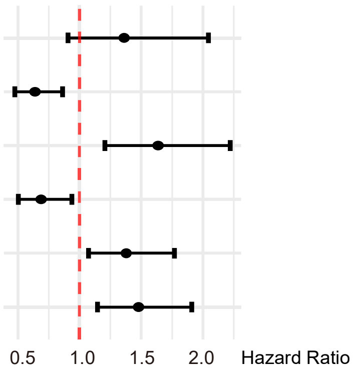

B

Multivariate Cox __TCGA-UCEC

Cell Population	HR (95% CI)	p−value
Angio−Mac	1.19 (0.72−1.95)	0.4984
IFN−Mac_CXCL9	0.64 (0.39−1.04)	0.0709
Memory B	0.67 (0.42−1.06)	0.0881
Atypical memory B	1.96 (1.00−3.83)	0.0497
TFs for Atypical memory B	1.25 (0.75−2.06)	0.3929

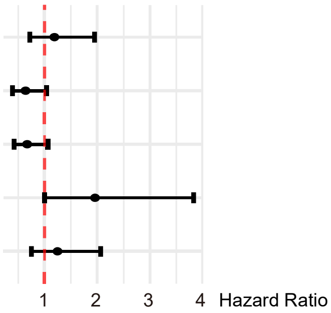

C

Multivariate Cox __TCGA-CESC

Cell Population	HR (95% CI)	p−value
Angio−Mac	2.93 (1.06−8.14)	0.0385
IFN−Mac_CXCL9	0.43 (0.20−0.89)	0.0239
NFKB1	1.99 (1.09−3.62)	0.0244
Memory B	0.31 (0.12−0.78)	0.0131
Atypical memory B	3.19 (1.15−8.83)	0.0253
TFs for Atypical memory B	2.26 (1.12−4.59)	0.0236

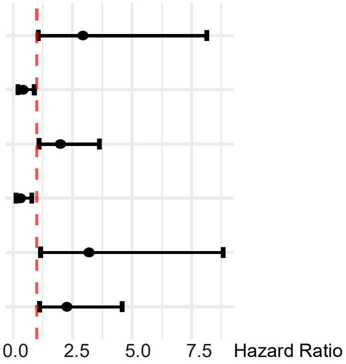

Figure S16. Multivariate Cox regression analysis of specific cell subsets and gene signatures adjusting for age, stage, and grade in tubo-ovarian (A), endometrial (B) and cervical (C) cancers using the TCGA database.

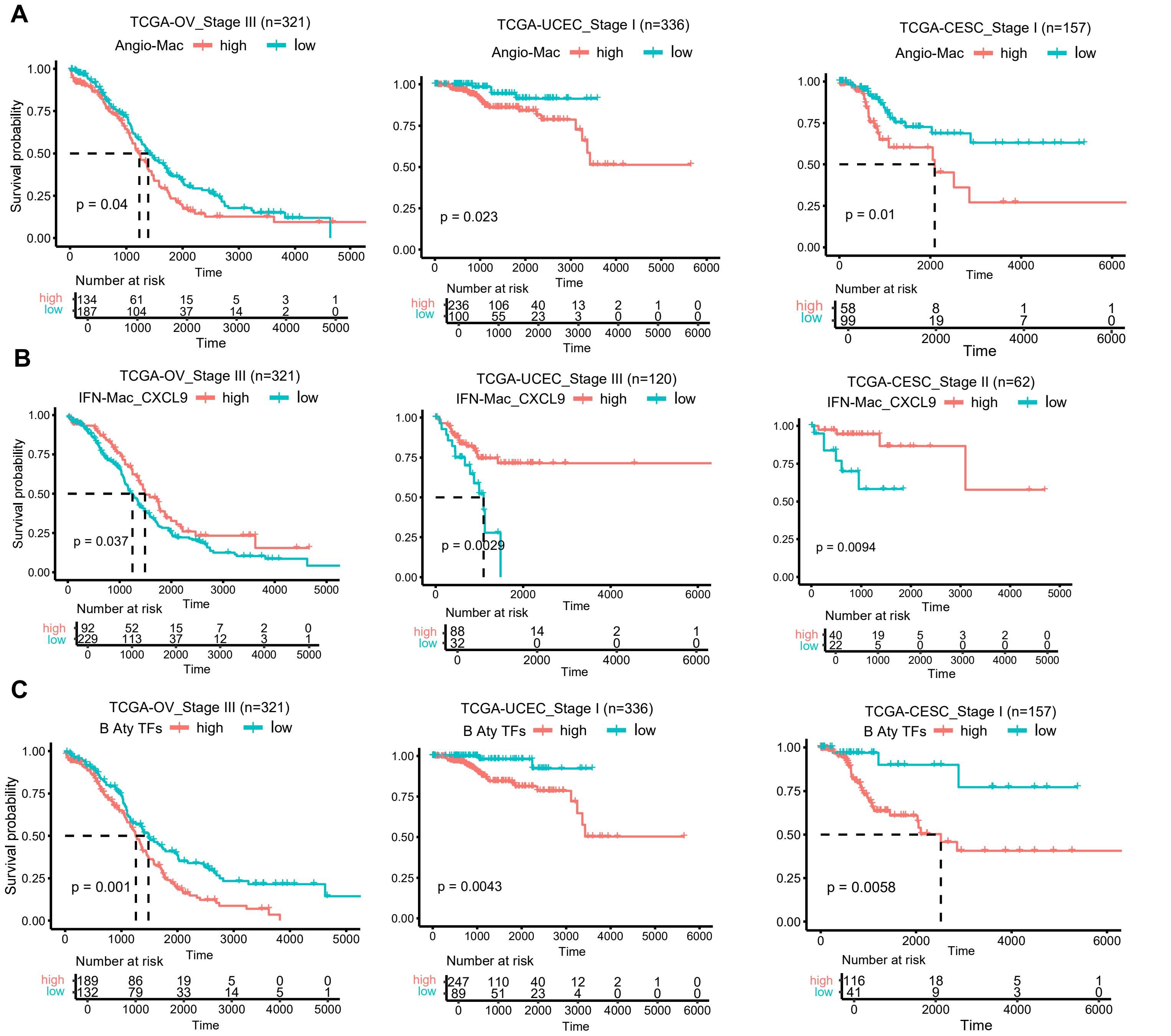

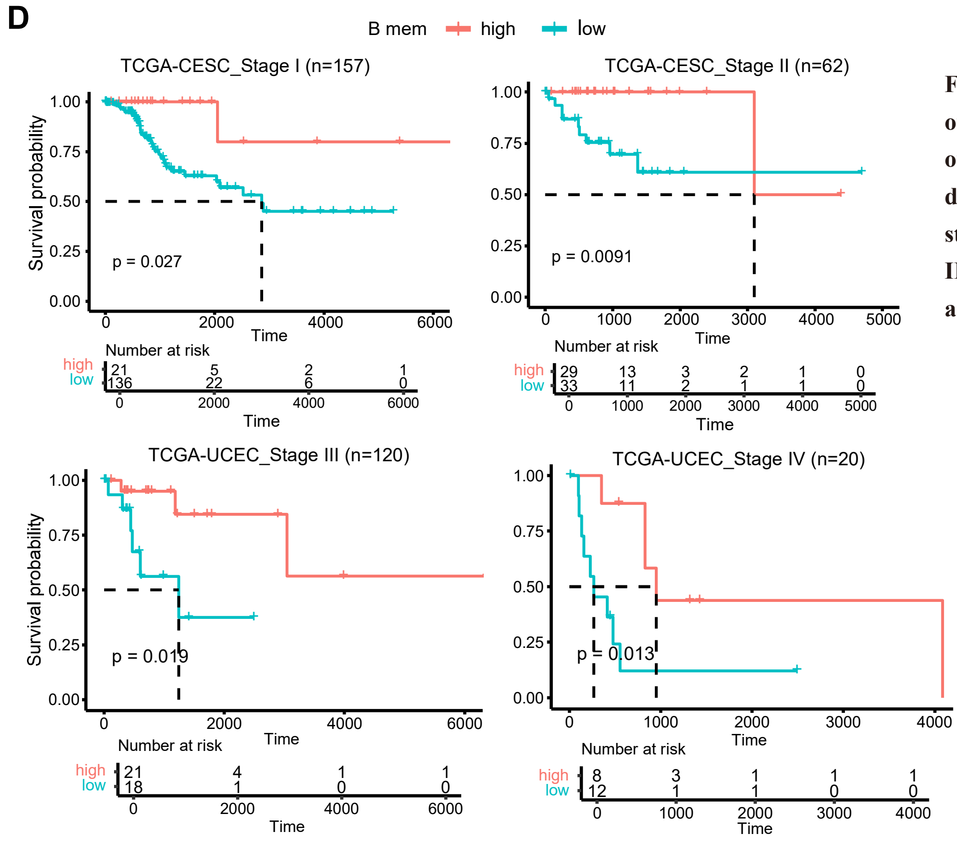

Figure S17. Kaplan-Meier curves with risk tables for overall survival generated from TCGA cohorts of ovarian, endometrial and cervical cancer with different stages, demonstrating significant prognostic stratification based on gene signature of Angio-Mac (A), IFN-Mac_CXCL9 (B), major transcription factors for atypical memory B cells (C) and memory B cells (D).
