# Supplementary material for: Single‐cell landscape of the tumour immune microenvironment in human gynaecologic malignancies
Source: Clin Transl Med. 2025 Nov 23;15(11):e70538. doi: 10.1002/ctm2.70538 (PMC12640613; doi:10.1002/ctm2.70538)
Supplement: Supplementary file 2 — Supporting Information [file CTM2-15-e70538-s003.pdf]

Supplemental Methods

A detailed breakdown of the samples by stage and type

Tubo-ovarian: 32 late-stage, 5 early-stage, 16 samples with no stage information available in the original source, one additional sample derived from an organoid model, and 33 non-malignant samples for comparison; 2) Endometrium: 3 late-stage, 5 early-stage and 7 non-malignant; 3) Cervical: 4 malignant samples with no stage information available in the original source and 5 non-malignant samples.

Robustness checks

We performed subsampling-based robustness checks by randomly retaining 90% of cells across 50 runs. For each run, we reclustered the cells on the Harmony embedding and compared against our previous clustering using Adjusted Rand Index (ARI) and per-cluster Jaccard indices.

Calculation of Ro/e (Ratio of observed to expected cell numbers)

To assess the distribution and preference of T cell subsets across different tissue conditions and tumor stages, the Ro/e (ratio of observed to expected cell numbers) [20,21] was calculated using the STARTRAC package (v. 0.1.0). The expected cell numbers for each cluster and stage were derived based on a chi-square test. Pairwise comparisons among groups were made using fisher's exact tests with FDR correction.

Slingshot pseudospatial trajectory analysis

To analyze cell lineage trajectories, Slingshot (v.2.7.0) 24 was used to infer developmental paths of cell clusters. For both CD8 and CD4 T cells, we excluded proliferating T cells and used the UMAP project as input. The naïve T cell population was selected as the start of the trajectory. To reduce computational demand, we randomly subsampled one-third of the cells for Slingshot analysis.

Inference of Transcription factor

For transcription factors (TFs) activity analysis, we employed the pySCENIC (v.0.12.1) workflow in python3.7. This method constructs regulatory networks and

identifies cell states from scRNA-seq data. The GRNBoost2 algorithm was used to calculate co-expression networks, and cisTarget was applied to identify regulons. The activity of each regulon in individual cells was quantified using AUCell. We utilized the ChIP-Atlas database (https://chip-atlas.org/) to acquire the binding sites of TFs to the genome in CD14+ monocytic cells and IGV (Integrative Genomic Viewer) was used for visualization of significant peaks (q-value < 0.01). RcisTarget (v.1.18.2) was applied for the identification of TF binding motifs enriched on signature genes of IFN-Mac_IFIT1.

Cell-cell interaction analysis

We analyzed the interactions between different immune cell subsets using the CellPhoneDB (v.4.0.0). This Python-based computational framework enables the analysis of cell-cell communication at the molecular level. For each cancer type (tubo-ovarian, endometrial, and cervical cancers), ligand-receptor pairs with significant interaction scores (p-value < 0.05) were extracted to assesses cell-cell communication patterns. The analysis was configured with the following key parameters: statistical significance was assessed through 1000 permutation iterations; genes were retained only if expressed in at least 10% of cells within any given cell subtype (threshold = 0.1). We employed the built-in statistical method to compute interaction scores and excluded subsampling to maintain full data integrity.

Hematoxylin and eosin (H&E) staining and immunohistochemistry (IHC) staining

The FFPE tissue samples were sectioned into 4-μm-thick slides, followed by deparaffinization in xylene and rehydrated through a graded ethanol series (100%, 95%, 85% and 75%) in deionized water. This preparation was applied for H&E, IHC, and mIHC staining. For H&E staining, we used the H&E Staining Kit (Servicebio, Wuhan, China). Briefly, the sections were stained with hematoxylin to visualized the nuclei, followed by eosin. staining. For IHC, antigen retrieval was performed by boiling the slides in antigen retrieval solution for 15 min. After blocking, the slides

were incubated overnight at 4°C with anti-CD68 primary antibodies (1:250, 76437, Cell Signaling Technology). Afterward, the sections were incubated with HRP-conjugated secondary antibodies (Gene Tech) for 30 min. Detection was achieved using DAB (Gene Tech). The stained slides were scanned with an OCUS Microscope Scanner (Grundium).
