# Supplementary material for: Single‐cell landscape of the tumour immune microenvironment in human gynaecologic malignancies
Source: Clin Transl Med. 2025 Nov 23;15(11):e70538. doi: 10.1002/ctm2.70538 (PMC12640613; doi:10.1002/ctm2.70538)
Supplement: Supplementary file 3 — Supporting Information [file CTM2-15-e70538-s002.pdf]

Table S1 to S10

Table S1. Information of the datasets used in the study

Reference	Cancer type	Cells	Patients	Samples	Stage (FIGO)	Tissue	Histology	Treatment	Data accession
[1]	tubo-ovarian	59,661	12	12	IC2, IIB, IIIB, IIIC, cancer free	ovary	7 HGSOC, 5 cancer free	treatment-naive	GSE184880
[2]	tubo-ovarian	32,079	1	6	IA, IIB, IIIA, IIIC	fallopian tube	high-grade serous fallopian tube carcinoma	3 treatment-naïve, 3 NACT	GSE191301
[3]	tubo-ovarian, endometrial	67,651	9	9	IA, IIB, IIIA, IIIC	ovary, endometrium	2 HGSOC, 1 serous, 6 endometrioid	treatment-naive	GSE173682
[4]	tubo-ovarian	52,121	5	5	IIIC, IV, IVA, IVB	omentum	HGSOC	chemotherapy	GSE154600
[5, 6]	tubo-ovarian	15,202	3	3	unknown	ovary	HGSOC	unknown	GSE158937
[7]	tubo-ovarian	49,916	8	8	unknown	ovary	HGSOC	treatment-naive	GSE217517

[8]	tubo-ovarian	45,115	5	10	IA, IIIC, IVB, cancer free	ovary, omentum, peritoneum	6 HGSOC,<br>1 HGSOC + clear cell carcinoma (mix),<br>3 cancer free	treatment-naive	E-MTAB-8107
[9]	tubo-ovarian	76,670	6	6	IIIC	ovary	HGSOC	treatment-naive	https://github.com/vicDRC/CCR_CD39study
[10]	tubo-ovarian	13,369	8	8	unknown	ovary, peritoneum, distance relapse site	2 HGSOC,<br>4 LGSOC,<br>1 Endometrioid,<br>1 SOC	6 treatment-naïve,<br>2 chemo/adjuvant therapy	GSE130000
[11]	tubo-ovarian	6,425	2	2	unknown	ovary, ascites	HGSOC	unknown	GSE140819
[12]	tubo-ovarian	8,199	/	1	unknown	organoid	HGSOC	/	GSE160755
[13]	tubo-ovarian	77,536	13	13	cancer free	fallopian tube	cancer free	/	GSE178101
[14]	tubo-ovarian	64,042	8	12	cancer free	fallopian tube	cancer free	/	GSE151214
[15]	endometrial	16,375	1	2	unknown	Lung	endometrial adenocarcinoma	1 treatment-naive,<br>1 NP137 treatment	GSE225689

[16]	endometrial	81,823	7	7	cancer free	endometrium	cancer free	/	GSE214411
[17]	cervical	20,938	1	2	unknown	cervix	1 SCC, 1 cancer free	treatment-naive	GSE168652
[18]	cervical	70,242	7	7	unknown	cervix	2 SCC, 1 ADC, 4 cancer free	treatment-naive	GSE208653

HGSOC: high-grade serous ovarian cancer; LGSOC: low-grade serous ovarian cancer; SOC: serous ovarian cancer; SCC: squamous cell carcinoma; ADC: adenocarcinoma; NACT: neoadjuvant chemotherapy

Table S2. Information of the independent validation corhorts

Reference	Cancer type	Cells	Patients	Samples	Stage (FIGO)	Tissue	Histology	Treatment	Data accession
[19]	endometrial	96,440	5	10	I	endometrium	5 endometrioid, 5 cancer free	treatment-naive	SRP349751
[20]	cervical	84,318	4	7	unknown	cervix	4 SCC, 3 cancer free	treatment-naive	E-MTAB-12305

References

1. Xu J, Fang Y, Chen K, Li S, Tang S, Ren Y, et al. Single-Cell RNA Sequencing Reveals the Tissue Architecture in Human High-Grade Serous Ovarian Cancer. Clin

Cancer Res. 2022; 28: 3590-602.
2. Shen Y, Ren Y, Chen K, Cen Y, Zhang B, Lu W, et al. The impact of neoadjuvant chemotherapy on the tumor microenvironment in advanced high-grade serous carcinoma. Oncogenesis. 2022; 11: 43.
3. Regner MJ, Wisniewska K, Garcia-Recio S, Thennavan A, Mendez-Giraldez R, Malladi VS, et al. A multi-omic single-cell landscape of human gynecologic malignancies. Mol Cell. 2021; 81: 4924-41.e10.
4. Geistlinger L, Oh S, Ramos M, Schiffer L, LaRue RS, Henzler CM, et al. Multiomic Analysis of Subtype Evolution and Heterogeneity in High-Grade Serous Ovarian Carcinoma. Cancer Res. 2020; 80: 4335-45.
5. Weber LM, Hippen AA, Hickey PF, Berrett KC, Gertz J, Doherty JA, et al. Genetic demultiplexing of pooled single-cell RNA-sequencing samples in cancer facilitates effective experimental design. Gigascience. 2021; 10: giab062.
6. Hippen AA, Falco MM, Weber LM, Erkan EP, Zhang K, Doherty JA, et al. miQC: An adaptive probabilistic framework for quality control of single-cell RNA-sequencing data. PLoS Comput Biol. 2021; 17: e1009290.
7. Hippen AA, Omran DK, Weber LM, Jung E, Drapkin R, Doherty JA, et al. Performance of computational algorithms to deconvolve heterogeneous bulk ovarian tumor tissue depends on experimental factors. Genome Biol. 2023; 24: 239.
8. Qian J, Olbrecht S, Boeckx B, Vos H, Laoui D, Etlioglu E, et al. A pan-cancer blueprint of the heterogeneous tumor microenvironment revealed by single-cell profiling. Cell Res. 2020; 30: 745-62.
9. Laumont CM, Wouters MCA, Smazynski J, Gierc NS, Chavez EA, Chong LC, et al. Single-cell Profiles and Prognostic Impact of Tumor-Infiltrating Lymphocytes Coexpressing CD39, CD103, and PD-1 in Ovarian Cancer. Clin Cancer Res. 2021; 27: 4089-100.
10. Kan T, Zhang S, Zhou S, Zhang Y, Zhao Y, Gao Y, et al. Single-cell RNA-seq recognized the initiator of epithelial ovarian cancer recurrence. Oncogene. 2022; 41: 895-906.
11. Slyper M, Porter CBM, Ashenberg O, Waldman J, Drokhlyansky E, Wakiro I, et al. A single-cell and single-nucleus RNA-Seq toolbox for fresh and frozen human tumors. Nat Med. 2020; 26: 792-802.
12. Wan C, Keany MP, Dong H, Al-Alem LF, Pandya UM, Lazo S, et al. Enhanced Efficacy of Simultaneous PD-1 and PD-L1 Immune Checkpoint Blockade in High-Grade Serous Ovarian Cancer. Cancer Res. 2021; 81: 158-73.
13. Ulrich ND, Shen Y-C, Ma Q, Yang K, Hannum DF, Jones A, et al. Cellular heterogeneity of human fallopian tubes in normal and hydrosalpinx disease states identified using scRNA-seq. Dev Cell. 2022; 57: 914-29.e7.
14. Dinh HQ, Lin X, Abbasi F, Nameki R, Haro M, Olingy CE, et al. Single-cell transcriptomics identifies gene expression networks driving differentiation and

tumorigenesis in the human fallopian tube. Cell Rep. 2021; 35: 108978.
15. Cassier PA, Navaridas R, Bellina M, Rama N, Ducarouge B, Hernandez-Vargas H, et al. Netrin-1 blockade inhibits tumour growth and EMT features in endometrial cancer. Nature. 2023; 620: 409-16.
16. Huang X, Wu L, Pei T, Liu D, Liu C, Luo B, et al. Single-cell transcriptome analysis reveals endometrial immune microenvironment in minimal/mild endometriosis. Clin Exp Immunol. 2023; 212: 285-95.
17. Li C, Guo L, Li S, Hua K. Single-cell transcriptomics reveals the landscape of intra-tumoral heterogeneity and transcriptional activities of ECs in CC. Mol Ther Nucleic Acids. 2021; 24: 682-94.
18. Guo C, Qu X, Tang X, Song Y, Wang J, Hua K, et al. Spatiotemporally deciphering the mysterious mechanism of persistent HPV-induced malignant transition and immune remodelling from HPV-infected normal cervix, precancer to cervical cancer: Integrating single-cell RNA-sequencing and spatial transcriptome. Clin Transl Med. 2023; 13: e1219.
19. Ren X, Liang J, Zhang Y, Jiang N, Xu Y, Qiu M, et al. Single-cell transcriptomic analysis highlights origin and pathological process of human endometrioid endometrial carcinoma. Nat Commun. 2022; 13: 6300.
20. Li C, Hua K. Dissecting the Single-Cell Transcriptome Network of Immune Environment Underlying Cervical Premalignant Lesion, Cervical Cancer and Metastatic Lymph Nodes. Front Immunol. 2022; 13: 897366.

Table S3. Information of the FFPE specimens used in the study

ID	Age	Tissue	Histology	Stage (FIGO)	OS (months)	Recurrence
HGSOC 01	59	ovary (left)	HGSOC	IIIB	/	/
HGSOC 02	57	ovary (right)	HGSOC	IIB	/	/
HGSOC 03	53	ovary (right)	HGSOC	IVB	/	/
Patient 1	63	ovary (left)	HGSOC	IIIC	31	yes
Patient 2	62	ovary (right)	HGSOC	IIIC	38	yes
Patient 3	43	ovary (right)	HGSOC	IIIC	16	unknown
Patient 4	69	ovary (left)	HGSOC	IV	7	unknown
Patient 5	52	ovary (left)	HGSOC	IIIC	26	yes
Patient 6	50	ovary (left)	HGSOC	IIIC	38	yes
Patient 7	59	ovary (left)	HGSOC	IIIC	≥60	no
Patient 8	45	ovary (right)	HGSOC	IIA	≥60	no
Patient 9	49	ovary (left)	HGSOC	IIA	≥60	no
Patient 10	55	ovary (left)	HGSOC	IIIC	≥60	no
Patient 11	47	ovary (right)	HGSOC	IIIC	≥60	no
Patient 12	53	ovary (left)	HGSOC	IIB	≥60	no

OS: Overall survival; HGSOC: high-grade serous ovarian cancer

Table S4. Signature genes of IFN-Mac_CXCL9 that differentially expressed in tumor and normal tissue

IFN-Mac_CXCL9 signatures upregulated in tumor	IFN-Mac_CXCL9 signatures upregulated in normal tissue
CXCL10, CXCL9, LAP3, IL4I1, CXCL11, GBP1, PSME2, GBP4, APOL3, HAPLN3, TYMP, TMSB10, FN1, CD40, ATF5, SLAMF7, STAT1, GBP5, TAP1, SERPING1, ATOX1, PRDX1, PSMB9, C15orf48, ENO1, UBE2L6, EPSTI1, PSMB8, BST2, HLA-DRB1, LY6E, ANXA2, IFI35, NUB1, HLA-DQA1, LILRB4, PSME1, FCGR3A, CAPG, SNX10, TNFSF13B, IFI6, TGM2, LDHA, SYNGR2, HLA-DQB1, YWHAH, PKM, PSMB2, CD74, SAMD9L, GSN, TUBB, IL32, MT2A, TPI1, RAB13, GRN, PGAM1, OAS2, APOL2, ISG15, PFKP, VIM, IFNGR2, IFIH1, IRF7, PML, OAS1, OAS3, IFIT3, MX1, GAPDH, HLA-F, RPS27L, HLA-A, PARP12, IFI44L, TMEM51, GALM, NR1H3, IFITM3, RSAD2, SMAD7, DDT, LGALS3BP, HLA-DRB5, IFIT2, CCL5, HLA-C, TNS3, B2M, GM2A, FMNL2, SPP1, CCL2, ID2, PARP10, APOBEC3A, IFI44, CYB5A, APOC1, C2, KCNMA1, IFI27, MMP14, APOE, C1QB	CLEC10A, HLA-DQA2, FCN1, CST3, CSF1R, NDUFA11, ARPC1B, EEF1B2, MOB1A, RPL28, RPL27, DOK2, CD36, CPVL, TIMP1, RPS5, PARL, RPS10, IFI30

Table S5. Signature genes of Angio-Mac that differentially expressed in tumor and normal tissue

Angio-Mac signatures upregulated in tumor	Angio-Mac signatures upregulated in normal tissue
C15orf48, IFNGR2, TXN, DDX60L, PLK3, FNDC3B, TNIP1, NBN, GRINA, SLC6A6, IVNS1ABP, RBM17, MAP4K4, PSTPIP2, ASAP1, CPD, HIPK2, MYO1G, S100A10, ATXN1, LRRFIP2, PIK3R5, SUSD6, PELI1, AFF4, NCOR2, RNF145, IL1RN, SPAG9, ETV6, CXCL8, PTPN12, KLF10, VEGFA, NFKBIA, FOXO3, HCK, PLEKHB2, ZMIZ1	SERPINB2, THBS1, VCAN, EREG, LGALS2, FCN1, CD300E, CCL20, IL1A, CXCL2, CFP, NLRP3, PTGER2, LYZ, CXCL3, LDLR, CYP1B1, ATP2B1, THAP2, TIMP1, CD36, SERPINB9, GPCPD1, CCRL2, SERPINB8, CLEC12A, CRIP1, CTNNB1, PHLDA1, STXBP2, WTAP, SAMSN1, TNFAIP8, TFDP1, EIF1B, INSIG1, PNPLA8, CD55, RPL17, FAM107B, DUSP6, MPEG1, OLR1, TKT, EEF1G, PLAUR, BIRC3, BACH1, B4GALT1, IFI30, EZR, STX11, BCL2A1, ARL5B, CHMP1B, C9orf72, AP1S2, PNRC1, RPL34, CHP1, BTG1, ATP1B3, RPL36A, CTSS, AOAH, SPATA13, CDKN1A, CREM, RPL39, RPS13, PABPC1, CYCS, CD302, SRGN, CPVL, YBX3, TGIF1, PFDN5, SFPQ, EMD, RPL35A, RPL26, EIF4E, EIF3L, RPS28, HBP1, EIF3M, ELL2, RPS9, PPP1CB, RPS27, RPS24, RPL18A, RPL32, PTPRE, EIF1, SERPINB1, RPS16, RPLP1, RPS8, BCL3, DDX21, SOCS3, RPS15A, ELF1, RPS14, ATF4, EIF2S3, RPS26, CCNH, RPL37, MAT2A, NR4A3, EEF2, MYADM, RPL28, UBA52, RPL27, OTUD1, MCL1, RPL11, GLIPR1, RPL30, RPS12, RPL41, RPL23, EIF3E, PRNP, PNRC2, SRSF6, FAU, RPL13, RPS7, RPL18, RPL10, RPS11, RPLP2, C5AR1, EIF4B, RPS3A, EEF1D, RPL29, RPS15, TMEM123, RPL24, CNIH1, EIF4G2, SYAP1, NDEL1, RPL7,

	RPL22, NACA, RPS29, BTF3, RPS21, RASSF3, RPS10, RPL4, RPL37A, SUB1, COMMD6, DUSP2, RPL9, CRTAP, NFE2L2, RPL19, RPS18, RPL31, SRSF3, CFD, RPL38, RPL23A, SELL, EEF1A1, C16orf72, RPL36

Table S6. GO enrichment analysis for NFKB1 targets

ID	Description	pvalue	qvalue	geneID
GO:2001234	negative regulation of apoptotic signaling pathway	0.000135	0.012042	CFLAR, SOD2, CD44, THBS1
GO:0032102	negative regulation of response to external stimulus	0.000153	0.012042	NLRP3, NFKB1, SERPINB9, RIN3, THBS1
GO:0032695	negative regulation of interleukin-12 production	0.000199	0.01208	NFKB1, THBS1
GO:0036293	response to decreased oxygen levels	0.000364	0.014381	ZEB2, CFLAR, SOD2, THBS1
GO:0010884	positive regulation of lipid storage	0.000407	0.014381	NFKB1, EHD1
GO:0030511	positive regulation of transforming growth factor beta receptor signaling pathway	0.00061	0.015054	ZEB2, THBS1

Table S7. Adjusted p-values comparing the distribution of T cell subsets across different stages

Cluster	Tubo-ovarian Cancer			Endometrial Cancer			Cervical Cancer
	Normal vs Early	Norm vs Late	Early vs Late	Normal vs Early	Norm vs Late	Early vs Late	Norm vs Cancer
CD8 Naive	0	0	2.24E-39	1.73E-53	1.91E-11	2.26E-14	2.52E-82
CD8 Tem	1.05E-64	0	2.26E-56	5.37E-10	0.116543	3.88E-05	1.93E-62
CD8 Trm	0	0	7.30E-45	0.820722	0.820722	0.820722	2.23E-41
CD8 Tcm	3.17E-62	1.20E-240	0.006858975	7.55E-11	4.59E-09	0.389081	9.19E-21
CD8 Tex	0	0	4.89E-49	7.91E-09	1.50E-23	0.001538	2.56E-16
CD8 Innate-like	9.50E-14	3.63E-27	0.005084506	7.13E-35	4.34E-38	1	7.20E-14
CD8 Temra	0.030969855	5.01E-38	0.000262932	0.383714	1.89E-10	1.46E-05	5.63E-05
CD8 Prol	0.127481878	3.43E-05	0.805301518	0.001069	0.000546	0.906316	0.178954
CD4 Tcm	0	0	0.001772	7.37E-42	7.59E-48	0.531323	7.29E-64
TNFRSF9 Treg	2.50E-320	0	2.02E-06	9.57E-05	1.74E-23	6.46E-12	1.17E-35
CD4 Naive	0.06287447	0.00511098	0.564658	3.26E-34	0.000555	6.90E-26	6.06E-33
Th1-like	2.32E-158	3.11E-266	1.64E-11	0.940215	7.54E-08	1.30E-09	5.12E-18
IFIT3 Treg	1.74E-139	4.32E-236	4.99E-06	0.834887	1	0.834887	0.000359

Table S8. Signature genes of CD8 Trm that differentially expressed in early-stage and late-stage tubo-ovarian cancer

CD8 Trm signatures upregulated , in early-stage tubo-ovarian cancer	CD8 Trm signatures upregulated , in late-stage tubo-ovarian cancer
VMP1, IFI27, IFI44L, IFI44, SLFN5, RBPJ, MX2, NT5C3A, UPF2, ISG15, MAF, SAMD9, USP18, RNF213, SAMD9L, IFI16, ARID1B, ODF2L, IFIT1, SETX, APOL6, DDX60L, ZRANB2, SAT1, TYMP, TRIM56, IFIT3, SPATS2L, FAM118A, IRF3, PTPRA, N4BP1, IKZF3, NLRC5, GPBP1, GNB1, EPSTI1, GBP2, XAF1, PARP14, GBP1, KMT2A, CD2, C6orf62, CTSS, DDX17, TRIM38, STAT2, NUB1, UBALD2, RSAD2, CD48, DDX60, MLLT6, ARPC1B, VPS13C, LPIN2, GNAI2, CASP4, ACTN4	IFITM1, PFN1, CD74, MIF, ACTB, GABARAP, SH3BGRL3, S100A10, CISH, SOCS1, JUN, PSMB10, IFITM3, TXNIP, LY6E, COTL1, S100A4, ZYX, CORO1B, NFKBIA, EMP3, OTUB1, UBE2L6, ALOX5AP, GNB2, GPSM3, CORO1A, GLIPR2, CLIC1, HLA-DRB1, ITGA4, BAX, PSME2, LTB, BCAP31, TBCB, NAPA, HLA-DPA1, GSTK1, PKM, PIM1, VCP, GNG5, SKAP1, CCND3, PRR13, PPP1CA, ARF5, HCLS1, PSME1, SLC9A3R1, SHISA5, ANXA11, ADGRE5, CAP1, CXCR3, VAMP8, PGAM1, HLA-DQA1, BHLHE40, MVP, SELPLG, TRAPPC1, ANXA6, RALY, LRRFIP1, PCBP1, WDR1, ACTG1, PSMB9, RAC1, SIT1, PPP1R18, IFITM2, NUCB1, HLA-DRA, LAT, IFI35, YWHAB, TSC22D4, RAC2, THRAP3, WAS, TSPO, INPP4B, EIF5A, OPTN, TMSB10, ITGB7, ENO1, PYURF, PPP4C

,

Table S9. Signature genes of TNFRSF9 Treg that differentially expressed in early-stage and late-stage tubo-ovarian cancer

TNFRSF9 Treg signatures upregulated , in early-stage tubo-ovarian cancer	TNFRSF9 Treg signatures upregulated , in late-stage tubo-ovarian cancer
MAF, IL2RG, VMP1, NDUFC2, FAM89B, TMBIM4, NDUFB8, ABHD17A, ISG15, DYNC1I2, RNF213, MXD4, ZNRF1, ENTPD1, MBD2, GNAI2, BRI3, RASGRP1, N4BP1, CASK, BLOC1S1, NDUFB1, NABP1, BOD1L1, COMMD7, MAP2K2, EPSTI1, ITGAL, SPATS2L, EIF3J, CKLF, CBX3, STAT1, TAF10, EFHD2, PSMB4, CD2, PET100, SAMD9L, SAMD9, HEBP2, ACTN4, SAT1, CASP4, PTPRA, MSI2, HNRNPD, APOL6, LIMS1, CD3D, NDUFA3, DYNLL1, TMCO1, DDX17, CDV3, TBL1XR1, TYMP, ARPC1B, MLLT6, CASP8, XAF1, CTSS, NAMPT, PREX1, UGP2, FMNL1, PRPF4B, HLA-F, CHURC1, ARPP19, IFI6, GADD45A, UBL5, SUB1, ALDOA, TBCA, ACTR2, GBP4	CD74, PFN1, GABARAP, ACTB, MIF, LTA, CISH, FOXP3, PKM, PPDPF, HLA-B, GAPDH, TPI1, LSP1, HLA-DRB1, PSMB10, SYNGR2, SELPLG, RHOA, CD3E, TNFRSF4, VCP, CD7, CORO1B, SQSTM1, PCBP1, IL2RA, PIM2, AKIRIN2, BATF, ARF5, HSPB1, UCP2, PGAM1, DEF6, CD82, SH2D2A, WDR1, CMTM3, DAZAP2, TUBB, RHBDD2, LGALS1, HLA-DQA1, SELL, LCP2, GRSF1, HLA-DPA1, NCF4, PSAP, SOCS1, IL10RA, GPI, OTUB1, GNB2, PELI1, OS9, CXCR3, PRR13, POLR2E, PPP1CA, JAK1, RAC2, ACTG1, ANXA11, UBE2L6, NCOR1, GATA3, HCLS1, AQP3, CDC37, AUP1, SHISA5, HNRNPLL, SF3B2, HM13, FKBP8, XRCC6, PSME2, RFTN1, RNF167, CORO1A, LAYN, DNPH1, TNFRSF1B, STAM, TNIP1, LTB, TMED9, EMP3, SLC9A3R1, ACP5, PPP1R18, BCAP31, SIT1, FKBP1A, TPM3, ENO1, UBE2D2, LCK, WSB1, LY6E, RHOH, EWSR1, RNF187, HLA-DQB1, PPP2R1A, LBH, PHB2, RCSD1, WDR83OS, CLTB, RHOG, AP2M1, LIMD2, IMP3, SLAMF1, NDUFAF3, PBXIP1, KHDRBS1, PSMF1, IL32, SKAP1, TMOD3, ZAP70, ANKRD10, RALY, ANXA6, TNFRSF18, LAT, CLIC1, HERPUD1, CAP1, MRPS6, TBCB, ARL6IP5, CTSB, NDFIP1, PPP4C, TADA3, PPM1G, CTSC, CD58, MVP, SH3KBP1, SCAMP2, LAMP1, RAB7A, VASP, RAD21, RPN2, SASH3, RAC1, EDF1, COX6A1, S100A4, COX8A, SDF4, EID1, CTSA, WAS, MDH2, COX5A, CD27, DBNL, BRK1, ADRM1, NUCB1, BSG, UBC, MYL6, EIF5A, HLA-A, HNRNPA2B1, TMEM179B

Table S10. GO enrichment analysis for ZBTB20 targets

ID	Description	pvalue	qvalue	geneID
GO: 0043161	proteasome-mediated ubiquitin-dependent protein catabolic process	4.91E-15	2.13E-11	FAF1, MAN1A2, RNF187, XPO1, COMMD1, PELI1, UBXN4, SPOPL, PSMD14, DNAJC10, CUL3, STT3B, CTNNB1, ARIH2, FHIT, PCNP, TBL1XR1, UBXN7, FBXL5, CLOCK, UBE2D3, USP38, FBXW7, SH3RF1, FBXL17, FEM1C, FBXO38, FBXW11, FAF2, AKIRIN2, FBXL4, ASCC3, MAN1A1, GNA12, ZNRF2, ANKIB1, SMURF1, CUL1, AGAP3, TBL1X, USP9X, MTM1, RNF122, WWP1, RNF19A, UBQLN1, FBXO3, WAC, CUL2, SIRT1, FBXW4, LRRK2, UBE2N, RNF34, TRIM13, FBXL3, HECTD1, FBXO33, KLHDC2, PSEN1, ATXN3, PPP2R5C, ARIH1, USP7, SIAH1, AMFR, WWP2, FBXL20, TLK2, PSMD12, USP14, ITCH, TRPC4AP, GIPC1
GO: 0006470	protein dephosphorylation	1.21E-09	5.86E-07	MTOR, PPP2R5A, DUSP10, PPP1CB, PPM1B, PPP3R1, PPP1R2, HTT, PPM1K, PPP3CA, PPP2CA, FBXW11, DUSP22, PTPRK, GNA12, MTM1, PPP2R2A, SWAP70, PPP6R3, PTPRB, PPTC7, PPP1CC, PTPN11, SPPL3, PPM1A, PPP2R5E, PPP2R5C, SSH2, PPM1D, PPP4R1, PTPN2, ROCK1, PPP1R16B, PTPN1, PTBP1, PTPRS, MTMR3
GO: 0016049	cell growth	1.54E-08	4.24E-06	SLC25A33, MTOR, CDC42, CDC73, ADAM17, PUM2, ITSN2, SERTAD2, EPB41L5, TGFBR2, GOLGA4, CTNNB1, IP6K2,

				ARIH2, FOXP1, ALCAM, RYK, ZNF639, EIF4G1, RUFY3, CAMK2D, JADE1, RICTOR, PPP2CA, WASF1, AUTS2, SMURF1, MTPN, RBBP7, CDKL5, RPS6KA3, USP9X, JADE3, NLGN3, SDCBP, SMARCA2, C9orf72, ABL1, USP47, SPAG6, SUPV3L1, PLCE1, ZFYVE27, CDKN1B, DIP2B, RAB21, CHPT1, RB1, NIN, INO80, SIN3A, PAFAH1B1, MAP2K4, SPAG9, SMAD4, MEX3C, BCL2, SLC23A2, ITCH, ADNP, PTPRS, URI1, APP
GO: 0031098	stress-activated protein kinase signaling cascade	3.19E-08	7.69E-06	GADD45A, MAPKAPK2, DUSP10, EIF2AK2, MAP4K4, ATF2, RNF13, DLG1, RELL1, NFKB1, SH3RF1, PTGER4, DUSP22, RIPK1, MAP3K5, MAP3K4, HIPK2, XIAP, STK26, RB1CC1, LYN, SDCBP, RIPK2, HIPK3, MAPK8, PDCD4, LRRK2, FOXO1, CCDC88C, IGF1R, CYLD, PAFAH1B1, MAP2K4, NCOR1, PHLPP1, ITCH, MAPK1, APP
GO: 0016055	Wnt signaling pathway	6.45E-08	1.33E-05	CTNNBIP1, CDC42, RNF220, CDC73, STRN, PPM1B, USP34, ZEB2, TTC21B, CCNYL1, CUL3, RAB5A, CTNNB1, RHOA, KPNA1, RYK, TBL1XR1, NFKB1, JADE1, OTULIN, CSNK1G3, PPP2CA, FBXW11, RPS12, LATS1, CDK14, SMURF1, HBP1, TBL1X, ZBTB33, XIAP, UBR5, TLE1, SPIN1, ABL1, USP47, BAMBI, CCNY, TNKS2, FBXW4, FAM53B, LRRK2, FOXO1, UBAC2,

				PPM1A, PSEN1, CCDC88C, CSNK1G1, TLE3, LRRK1, SIAH1, CYLD, AMFR, PLCG2, RNF138, MBD2, ADNP, APP
GO: 0007249	canonical NF-kappaB signal transduction	1.01E-07	1.52E-05	MIER1, TRAF5, PPM1B, REL, PELI1, SLC20A1, STAT1, CFLAR, CTNNB1, RHOA, TFRC, DHX15, NFKB1, NDFIP1, FBXW11, RIPK1, TAB2, ESR1, CUL1, TAB3, XIAP, RIPK2, TLE1, ABL1, TRAF6, SIRT1, DDX21, IFIT5, ERC1, TBK1, ZDHHC17, UBE2N, TRIM13, PELI2, PPM1A, TRAF3, RORA, ZFAND6, AKAP13, PLCG2, VAPA, ROCK1, SLC44A2
GO: 0051403	stress-activated MAPK cascade	1.06E-06	8.50E-05	GADD45A, MAPKAPK2, DUSP10, EIF2AK2, MAP4K4, ATF2, DLG1, RELL1, NFKB1, SH3RF1, PTGER4, DUSP22, RIPK1, MAP3K5, MAP3K4, HIPK2, XIAP, RB1CC1, SDCBP, RIPK2, HIPK3, MAPK8, PDCD4, LRRK2, FOXO1, IGF1R, CYLD, PAFAH1B1, MAP2K4, NCOR1, PHLPP1, ITCH, MAPK1, APP
GO: 0007254	JNK cascade	1.47E-06	0.000112	GADD45A, DUSP10, MAP4K4, ATF2, NFKB1, SH3RF1, PTGER4, DUSP22, RIPK1, MAP3K5, HIPK2, XIAP, RB1CC1, SDCBP, RIPK2, HIPK3, MAPK8, PDCD4, LRRK2, IGF1R, CYLD, PAFAH1B1, MAP2K4, NCOR1, PHLPP1, ITCH, APP

GO: 0031929	TOR signaling	2.39E-06	0.000159	MTOR, AKT3, CUL3, PIK3CA, GOLPH3, RICTOR, FNIP1, SESN1, GNA12, MIOS, RHEB, USP9X, MTM1, YWHAZ, C9orf72, SYK, SESN3, SIK3, WAC, SIRT1, TBK1, UBE2N, HIF1A, ATXN3, USP7, USP32, NPC1
GO: 0071559	response to transforming growth factor beta	6.45E-06	0.000361	ZFYVE9, ADAM17, EPB41L5, ZEB2, CFLAR, TGFBR2, NFKBIZ, SKIL, SMAD5, NR3C1, DUSP22, PTPRK, LATS1, SMURF1, HIPK2, RBBP7, USP9X, SDCBP, ABL1, BAMBI, SIRT1, USP15, CHST11, ARID4A, PPM1A, FUT8, SPRED1, RNF111, SIN3A, FURIN, IGF1R, ZFHX3, STAT3, ROCK1, SMAD2, SMAD4, NFATC1, GIPC1
GO: 0045739	positive regulation of DNA repair	9.48E-06	0.000483	ARID1A, DHX9, EPC2, RIF1, INO80D, TOP2B, PBRM1, UIMC1, ARID1B, PHF10, FANCB, SMARCA2, FAM168A, NFRKB, SIRT1, ARID2, UBE2N, EP400, INO80, MBTD1, SMCHD1, EYA2
GO: 0038202	TORC1 signaling	2.22E-05	0.000908	MTOR, CUL3, FNIP1, SESN1, MIOS, RHEB, YWHAZ, C9orf72, SYK, SESN3, SIK3, WAC, TBK1, UBE2N, ATXN3, USP7, USP32, NPC1
GO: 0030307	positive regulation of cell growth	4.19E-05	0.001455	SLC25A33, MTOR, CDC42, ADAM17, PUM2, ITSN2, GOLGA4, ZNF639, EIF4G1, RUFY3, RICTOR, SMURF1, MTPN, CDKL5, RPS6KA3, SDCBP, USP47,

				SUPV3L1, ZFYVE27, INO80, PAFAH1B1, BCL2, SLC23A2, ADNP
GO: 0038061	non-canonical NF-kappaB signal transduction	0.00021	0.004869	AGO3, EIF2AK2, PPM1B, REL, RHOA, NFKB1, NR3C2, RIPK1, TAB2, TAB3, TNFRSF10A, RC3H2, TRAF6, PDCD4, PPM1A, DICER1, UACA, CYLD, AMFR, NFAT5, APP
GO: 0097396	response to interleukin-17	0.000324	0.006569	TRAF5, NFKBIZ, NFKB1, TRAF6, STAT3, IL17RA
